# Angong Niuhuang Wan ameliorates LPS-induced cerebrovascular edema by inhibiting blood‒brain barrier leakage and promoting the membrane expression of AQP4

**DOI:** 10.3389/fphar.2024.1421635

**Published:** 2024-08-01

**Authors:** Bo-Tong Liu, Quan Li, Kai Sun, Chun-Shui Pan, Xin-Mei Huo, Ping Huang, Li Yan, Qi-Hua He, Li-Jun Zhong, Yuan Wang, Meng-Lei Hu, An-Qing Li, Ying-Qian Jiao, Shuang Zhang, Xiao-Yi Wang, Jian Liu, Jing-Yan Han

**Affiliations:** ^1^ Department of Integration of Chinese and Western Medicine, School of Basic Medical Sciences, Peking University, Beijing, China; ^2^ Tasly Microcirculation Research Center, Peking University Health Science Center, Beijing, China; ^3^ The Key Discipline for Integration of Chinese and Western Basic Medicine (Microcirculation) of the National Administration of Traditional Chinese Medicine, Beijing, China; ^4^ Key Laboratory of Stasis and Phlegm, State Administration of Traditional Chinese Medicine of the People’s Republic of China, Beijing, China; ^5^ Beijing Microvascular Institute of Integration of Chinese and Western Medicine, Beijing, China; ^6^ State Key Laboratory of Natural and Biomimetic Drugs, Peking University, Beijing, China; ^7^ Peking University Medical and Health Analysis Center, Peking University, Beijing, China

**Keywords:** blood-brain barrier, cerebrovascular edema, AQP4, PKC-α, VE-cadherin

## Abstract

**Introduction:**

Angong Niuhuang Wan (AGNHW), developed during the Qing dynasty (18th century) for the treatment of consciousness disturbances caused by severe infections, has been used to treat brain edema caused by ischemia‒reperfusion. However, it remains unclear whether AGNHW can ameliorate vascular-origin brain edema caused by lipopolysaccharides (LPS). This study explored the ameliorative effects of AGNHW on LPS-induced cerebrovascular edema in mice, as well as the potential underlying mechanisms.

**Methods:**

A cerebrovascular edema model was established in male C57BL/6N mice by two intraperitoneal injections of LPS (15 mg/kg), at 0 and 24 h. AGNHW was administered by gavage at doses of 0.2275 g/kg, 0.455 g/kg, and 0.91 g/kg, 2 h after LPS administration. In control mice, normal saline (NS) or AGNHW (0.455 g/kg) was administered by gavage 2 h after intraperitoneal injection of NS. The survival rate, cerebral water content, cerebral venous FITC-dextran leakage, Evans blue extravasation, and expression of vascular endothelial cadherin (VE-cadherin), zonula occludens-1 (ZO-1), claudin-5, phosphorylated caveolin-1 (CAV-1), and cytomembrane and cytoplasmic aquaporin 1 (AQP1) and aquaporin 4 (AQP4) were evaluated. The cerebral tissue phosphoproteome, blood levels of AGNHW metabolites, and the relationships between these blood metabolites and differentially phosphorylated proteins were analyzed.

**Results:**

AGNHW inhibited the LPS-induced decrease in survival rate, increase in cerebral water content, decrease in VE-Cadherin expression and increase in phosphorylated CAV-1 (*P*-CAV-1). AGNHW treatment increased the expression of AQP4 on astrocyte membrane after LPS injection. AGNHW also inhibited the LPS-induced increases in the phosphorylation of 21 proteins, including protein kinase C-α (PKC-α) and mitogen-activated protein kinase 1 (MAPK1), in the cerebral tissue. Eleven AGNHW metabolites were detected in the blood. These metabolites might exert therapeutic effects by regulating PKC-α and MAPK1.

**Conclusion:**

AGNHW can ameliorate cerebrovascular edema caused by LPS. This effect is associated with the inhibition of VE-Cadherin reduction and CAV-1 phosphorylation, as well as the upregulation of AQP4 expression on the astrocyte membrane, following LPS injection.

## Graphical Abstract

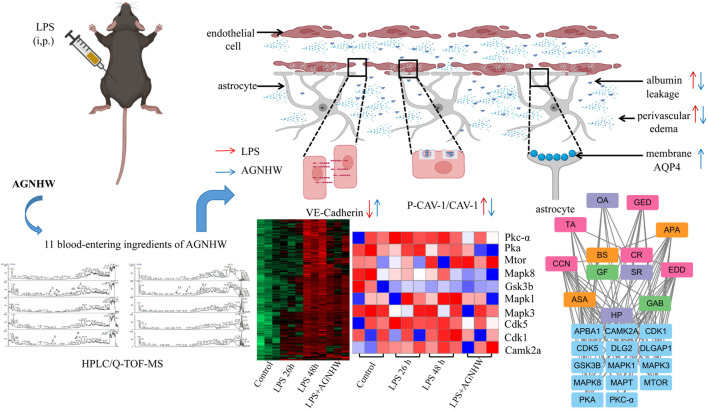

## 1 Introduction

Sepsis is a syndrome of physiological, pathological, and biochemical abnormalities caused by infection. Deaths from sepsis account for approximately one-fifth of annual global mortality (Rudd et al., 2020), with half of sepsis patients developing sepsis-associated encephalopathy (Mazeraud et al., 2020). Inflammatory response-induced blood‒brain barrier (BBB) damage and the resulting brain edema are important causes of sepsis-associated encephalopathy (Sekino et al., 2022).

Cerebral edema caused by sepsis is primarily of vascular origin (Li et al., 2019). Vascular-origin brain edema is caused by damage to the BBB and leakage of plasma outside microvessels (Wang et al., 2019), which in turn is mediated by paracellular leakage due to low expression of endothelial cell‒cell junction proteins and transcellular pathway leakage due to phosphorylation of caveolar proteins in endothelial cells (Pan et al., 2015). Water that has leaked to the outside of brain microvessels is absorbed through aquaporin 4 (AQP4) on astrocyte membranes (Zhang et al., 2022), and returns to the ventricles through AQP1 on the endothelial cells of the choroid plexus (Trillo-Contreras et al., 2019). The low expression of AQP4 on the membrane of astrocytes and of AQP1 on the endothelial cells of the choroid plexus may be the pathological basis for the slow absorption and reflux of edema (Papadopoulos and Verkman, 2007). However, no drugs have been clinically shown to both inhibit cerebral microvessel leakage and clear the leaked fluid (Perner et al., 2012).

AnGongNiuHuangWan (AGNHW) is a classic formula listed in Wu Jutong’s ‘Treatise on Febrile Diseases’, which was written during the Qing Dynasty (18th century). AGNHW is composed of 11 botanical Chinese medicines, including *Coptidis Rhizoma* (Ranunculaceae; *Coptis chinensis Franch*; Huanglian in Chinese)*, Gardeniae Fructus* (*Rubiaceae*; *Gardenia jasminoides* Ellis; Zhizi in Chinese)*, Scutellariae Radix* (*Lamiaceae*; *Scutellaria baicalensis* Georgi; Huangqin in Chinese)*, Curcumae Radix* (*Zingiberaceae*; *Curcuma wenyujin* Y.H. Chenet C. Ling; Yujin in Chinese)*, Artificialis Calculus Bovis* (*Bovis Calculus Artifactus*; Rengong Niuhuang in Chinese)*, Artificialis Moschus* (*Cervidae*; *artificial musk;* Rengong Shexiang in Chinese)*, Cinnabaris* (Zhusha in Chinese)*, Borneolum Syntheticum* (Bingpian in Chinese)*, Cornu Bubali* (Bovidae; Bubalus bubalis Linnaeus; Shuiniujiao in Chinese)*, Margarita* (Zhenzhu in Chinese)*,* and *Realgar* (Xionghuang in Chinese). It is used for the treatment of consciousness disturbances caused by severe infections. Previous studies have confirmed that AGNHW ameliorates brain edema caused by ischemia‒reperfusion (Chen et al., 2022), but whether it can also ameliorate vascular-origin brain edema caused by lipopolysaccharides (LPS), is not yet clear. In particular, it has not been determined whether AGNHW can inhibit the paracellular and transcellular pathway leakage of brain microvascular endothelial cells or promote the expression of AQP4 in astrocytes and AQP1 in the endothelial cells of the choroid plexus, and if so which metabolites of AGNHW absorbed into the blood play a role in this process.

## 2 Materials and methods

### 2.1 Reagents

In this study, we used standardized AGNHW from Tasly Pharmaceutical Co., Ltd. (Tianjin, China; batch number "20200507″). Each pill weighed 3 g and contained finely powdered *Coptidis Rhizoma* (Ranunculaceae; *C. chinensis*; Huanglian in Chinese) and *Curcumae Radix* (*Zingiberaceae*; *C. wenyujin* Y.H. Chenet C. Ling; Yujin in Chinese) (purchased from Henan Juren Traditional Chinese Medicine Decoction Pieces Co., Ltd.), *Gardeniae Fructus* (*Rubiaceae*; *G. jasminoides* Ellis; Zhizi in Chinese) and *Scutellariae Radix* (Lamiaceae; *S. baicalensis* Georgi; Huangqin in Chinese) (purchased from Anhui Jiahe Traditional Chinese Medicine Technology Co., Ltd.), *Artificialis Calculus Bovis* (*Bovis Calculus Artifactus*; Rengong Niuhuang in Chinese) (purchased from Sinopharm Holding Hubei Co., Ltd.), *Artificialis Moschus* (*Cervidae*; *artificial musk;* Rengong Shexiang in Chinese) (purchased from China Traditional Chinese Medicine Co., Ltd.), *Cinnabaris* (Zhusha in Chinese) (purchased from Hebei Yaoxing Limited Company), *Borneolum Syntheticum* (Bingpian in Chinese) (purchased from Yunnan Linyuan Flavors & Fragrances Co., Ltd), *Cornu Bubali* (Bovidae; *Bubalus bubalis* Linnaeus; Shuiniujiao in Chinese) (purchased from Fujian Zhongyi Pharmaceutical Co., Ltd), *Margarita* (Zhenzhu in Chinese) (purchased from Huzhou Zhenlu Biological Products Co., Ltd), *Realgar* (Xionghuang in Chinese) (purchased from Haozhou Purun Pharmaceutical Co., Ltd). The above powders were mixed, sieved, blended, and formed into honey pills (Supplementary Table S1).

### 2.2 Animals

Twelve-week-old male C57BL/6N mice weighing 24–26 g were obtained from Beijing Vital River Laboratory Animal Technology Co., Ltd. (license number: SYXK (Beijing) 2016–0044). The animals were housed in an SPF laboratory at a temperature of 20°C–26°C, humidity of 40%–70%, and a 12 h light/12 h dark cycle. The experiment followed the National Health Research Institute’s regulations on the use of experimental animals and was approved by the Laboratory Animal Ethics Committee of Peking University Health Science Center (PUIRB-LA2023528).

### 2.3 Drug administration and experimental groups

After 3 days of acclimatization, the mice were randomly divided into six groups: Sham + normal saline (NS), Sham + AGNHW (M), LPS + NS, LPS + AGNHW (L), LPS + AGNHW (M) and LPS + AGNHW (H). The mice in the LPS + NS, LPS + AGNHW(L), LPS + AGNHW(M), and LPS + AGNHW(H) groups were intraperitoneally injected with 15 mg/kg LPS at 0 and 24 h. The mice in the Sham + NS and Sham + AGNHW(M) groups received an equivalent volume of NS intraperitoneally at the same time points. Two hours after the second intraperitoneal injection, the mice in the Sham + NS and LPS + NS groups were gavaged with NS, while the Sham + AGNHW(M) (0.455 g/kg), LPS + AGNHW(L) (0.2275 g/kg), LPS + AGNHW(M) (0.455 g/kg), and LPS + AGNHW(H) (0.91 g/kg) groups received AGNHW by gavage. Cerebral tissues from the mice in the NS, LPS + NS 26 h, LPS + NS 48 h, and LPS + AGNHW (M) groups were subjected to phosphoproteomic analysis.

### 2.4 Survival rates

The cumulative survival rates of each group of mice over 72 h were statistically analyzed using GraphPad Prism 9.0 software (GraphPad Software, CA, USA).

### 2.5 Brain water content

Brain water content was measured as previously described (Hu et al., 2009). The brains were quickly divided into left and right cerebral hemispheres, and their initial weights were recorded (wet weight). Subsequently, the samples were dehydrated in an oven at 120°C for 48 h and then reweighed (dry weight). The percent water content was calculated using the following formula: ((wet weight - dry weight)/wet weight)*100.

### 2.6 Evaluation of BBB permeability

To determine albumin leakage, a noninvasive method was applied as previously described (Xu et al., 2009). In brief, following anesthesia, a cranial drill was used to carefully thin the skull of the mouse at the right parietooccipital cortex, which aligns with the edge of the MCA territory. Cerebral venules measuring between 35 and 45 μm in diameter and 200 μm in length were identified and selected with the aid of a fluorescence microscope (BX51WI, Olympus, Tokyo, Japan). Ten minutes prior to the observations, the mice received an intravenous injection of 50 mg/kg fluorescein isothiocyanate (FITC)-labeled dextran (70 kDa, Sigma‒Aldrich, MO, USA) via the femoral vein. The fluorescence signal was captured (excitation wavelength from 420 to 490 nm, emission wavelength at 520 nm) using an ultrasensitive CCD camera (USS-301, UNIQ Vision Inc., Santa Clara, CA, USA). The levels of FITC-labeled dextran in the venules (Iv) and surrounding interstitial areas (Ii) were quantified using ImageJ software (Bethesda, MD, USA). The leakage of FITC-labeled dextran was expressed as the ratio of Ii to Iv.

Evans blue dye (4%, Sigma‒Aldrich, St. Louis, MO, USA) dissolved in 0.9% saline (2 mL/kg, Sigma‒Aldrich, MO, USA) was administered intravenously into the right femoral vein. After 3 h, the mice underwent transcardial perfusion, and brain tissues were extracted for analysis of Evans blue content using a small-animal PET scanner (excitation at 620 nm, emission at 680 nm, Perkin-Elmer, MA, USA). The tissues were then homogenized in 1 mL of 50% trichloroacetic acid and centrifuged; the supernatant was collected and subsequently diluted four times with ethanol. The fluorescence intensities were determined using a spectrophotometer (excitation at 620 nm, emission at 680 nm; Molecular Devices, CA, USA). The amount of extravasated Evans blue dye was quantified and reported in nanograms per ischemic hemisphere.

### 2.7 Establishment of the blood–brain barrier cell model

To establish a blood‒brain barrier (BBB) cell model, a coculture system of HA1800 normal human astrocytes and HCMEC/D3 brain microvascular endothelial cells was utilized. The astrocytes were cultured in specialized astrocyte medium, while the endothelial cells were cultured in DMEM containing 10% fetal bovine serum and 1% penicillin‒streptomycin. First, poly-L-lysine was used to coat the bottom of the wells. HA1800 astrocytes were seeded on the bottom of the wells at a density of 4 × 10^4^ cells/cm^2^. When the astrocytes reached 70% confluence, HCMEC/D3 brain microvascular endothelial cells were seeded on top at a density of 1 × 10^5^ cells/cm^2^. The coculture was maintained for 3°days in DMEM containing 10% fetal bovine serum and 1% penicillin‒streptomycin. The cells were cultured in an incubator at 37°C with 5% carbon dioxide.

### 2.8 Cell viability assay

Twelve hours before the assay, the culture medium was replaced with serum-free DMEM containing 1% penicillin‒streptomycin. The effects of curcolonol (CCN), alphitolic acid (APA), Germacr-1 (10)-ene-5,8-dione (GED), octanoic acid (OA), gardenolic acid B (GAB), 4,10-epizedoarondiol (EDD), and asiatic acid (ASA) (at concentrations of 10^−4^, 10^−5^, and 10^−6^ mol/L) on the viability of cocultured brain microvascular endothelial cells and astrocytes were assessed after 24 h of incubation to determine the appropriate drug concentrations. Cell viability was measured using a CCK-8 assay kit, and the absorbance at 450 nm was read using a multifunctional microplate reader.

### 2.9 Cell experiment grouping, modeling, and administration

The cells were divided into the following groups: control, LPS, LPS + ASA, LPS + APA, LPS + EDD, LPS + GED, LPS + OA, LPS + GAB, and LPS + CCN. The experimental procedures for each group were as follows. In the control group, the cells were cultured normally and treated with PBS at 0 h. After 26 h, DMSO (at a final concentration of 0.01%) was added. In the LPS + DMSO group, the cells were cultured normally and treated with LPS (1 μg/mL) for 0 h. After 24 h of stimulation, the medium was replaced, and LPS (1 μg/mL) was added again. Two hours after the second LPS stimulation, DMSO (0.01% final concentration) was added. In the LPS + ASA, LPS + APA, LPS + EDD, LPS + GED, LPS + OA, LPS + GAB, and LPS + CCN groups, cells were cultured normally and treated with LPS (1 μg/mL) for 0 h. After 24 h of stimulation, the medium was replaced, and LPS (1 μg/mL) was added again. Two hours after the second LPS stimulation, ASA, APA, EDD, GED, OA, GAB, and CCN (each dissolved at a concentration of 10^−5^ mol/L in DMSO) were added.

### 2.10 Western blot analysis

Western blot analysis was performed as described previously (Huang et al., 2007). Protein concentrations were determined using the BCA assay. One hundred micrograms of protein from each sample was mixed with 10× sample buffer, boiled, and then loaded onto 10% SDS‒PAGE gels. Following electrophoresis, the separated proteins were transferred to nitrocellulose membranes (Hybond-C, Amersham Biosciences, USA). The membranes were then incubated at 4°C overnight with primary antibodies directed against β-actin, VE-Cadherin, ZO-1, Claudin 5, CAV-1, AQP4, AQP1, P-Tau (T231), Pan-Tau (Abcam, Cambridge, UK), *P*-CAV-1 (Tyr14, Cell Signaling, Beverly, Massachusetts, USA), and Na+/K + -ATPase (Immunoway, TX, USA). Subsequently, the membranes were washed with TBST and treated with horseradish peroxidase-conjugated secondary antibodies at a dilution of 1:5000 for 60 min at ambient temperature. The protein bands were visualized using enhanced chemiluminescence, and the optical density of each band was quantified with Image Lab software (Bethesda, MD, USA).

### 2.11 Immunofluorescence staining

Freshly frozen coronal tissues were cut into 10 μm slices using a cryostat (CM 1900, Leica, Bensheim, Germany). The slices were washed three times with PBS and blocked with 3% normal goat serum at room temperature for 30 min. The membranes were then incubated with primary antibodies diluted in PBS overnight at 4°C. The primary antibodies used included the following: anti-VE-cadherin (1:200, ab232515, Abcam, Cambridge, United Kingdom); anti-AQP4 (1:200, ab284135, Abcam, Cambridge, United Kingdom); anti-GFAP (1:200, ab207165, Abcam, Cambridge, United Kingdom); and anti-vWF (1:50, GTX28822, Gene Tex, CA, United States). On the following day, the brain sections were treated with DyLight Cy3-labeled goat anti-rabbit IgG (1:100, 5230-0359, SeraCare, MA, USA) and DyLight 680-labeled anti-rabbit IgG (1:100, 5230-0403, SeraCare, MA, USA) for 2 h at room temperature. Hoechst 33342 (1:50, H342, Dojindo, Kumamoto, Japan) was used to stain the nuclei. Finally, the sections were mounted, coverslipped, and imaged using a laser scanning confocal microscope (AXR, Nikon, Tokyo, Japan).

At 22 h after the last LPS administration, the culture medium was removed from the cocultured brain microvascular endothelial cells and astrocytes. The cells were washed with PBS and then fixed at room temperature with 4% paraformaldehyde for 30 min. After being washed with PBS three times, the cells were permeabilized with PBST containing 0.1% Triton X-100 at room temperature for 5 min. Following three PBS washes, the cells were blocked with goat serum at room temperature for 30 min. Primary antibodies were diluted at a ratio of 1:100 in primary antibody dilution solution, added to the wells, and incubated at 4°C overnight. The primary antibodies used included anti-AQP4, anti-N-Cadherin and anti-PKC-ɑ (Abcam, Cambridge, UK). On the following day, the plates were allowed to equilibrate to room temperature for 20 min. After three washes with PBS, fluorescent Cy3 and Dylight 647 secondary antibodies were diluted at a ratio of 1:100 in PBS and added to the plates, which were then incubated in the dark at 37°C for 2 h. After three washes with PBS, Hoechst 33342 diluted at a ratio of 1:50 was added, and the plates were incubated at room temperature for a further 10 min. After three PBS washes, the cells were replaced in fresh PBS and placed in a micro-lens dual spinning disk confocal high-content system (CV8000, YoKogawa, Tokyo, Japan). Twenty-five fields of view were captured per well (N = 6). The co-localization of N-Cadherin and AQP4 signals on astrocyte membranes were analyzed using Cell Pathfinder.

### 2.12 Phosphoproteome analysis

Mouse brain tissues were lysed in prechilled lysis buffer containing protease and phosphatase inhibitors (50 mg/500 μL). The protein concentration was measured using the Bradford method. Dithiothreitol (J&K Scientific, Beijing, China) and iodoacetamide (Sigma‒Aldrich, MO, USA) were added, followed by overnight incubation at 37°C with trypsin (Beyotime Biotechnology, Shanghai, China) in an ultrafiltration tube (10 kDa, 4 mL, Sartorius, Göttingen, Germany). Phosphorylated peptides were enriched using Ti-Immobilized Metal Affinity Chromatography (Ti-IMAC) (J&K Scientific, Beijing, China). The samples were then concentrated by rotary evaporation, redissolved, and analyzed by HPLC/Q-TOF-MS (U3000/Q Exactive HF, Thermo Fisher Scientific, Waltham, USA). The mass spectrometry data were analyzed using MaxQuant 1.5.8.3, with statistical analysis performed in Perseus 1.6.15. Phosphokinase activity was analyzed using posttranslational modification signature enrichment analysis (PTM-SEA) based on the PTMsigDB v2.0.0 kinase database (Krug et al., 2019). A protein‒protein interaction network was constructed with the STRING protein interaction database, and the core proteins in the interaction network were identified with Cytoscape v3.10.1.

### 2.13 AGNHW blood prototype and metabolite detection

To study the prototype metabolites of AGNHW absorbed into the blood and the metabolites metabolized by intestinal bacteria and the liver, mice were gavaged with 0.455 g/kg AGNHW. Serum was collected before administration and at 0.5, 1, 2, and 4 h postadministration and then analyzed using HPLC/Q-TOF-MS with retention times of 0–30 min in both positive-ion and negative-ion modes. Additionally, metabolites within AGNHW were identified through TCMIP (Xu et al., 2019) and MetFrag (Ruttkies et al., 2016). The predicted metabolites were analyzed using BioTransformer 3.0 (Djoumbou-Feunang et al., 2019; Wishart et al., 2022).

### 2.14 AGNHW blood metabolite target fishing and association study between blood metabolite targets and differentially phosphorylated proteins

Target fishing for AGNHW blood metabolites was conducted by using the Integrative Pharmacology-based Network Computational Research Platform of Traditional Chinese Medicine (TCMIP). The threshold for identifying chemical metabolites as exhibiting structural similarity was 0.6. The targets of AGNHW blood metabolites and the differentially phosphorylated proteins identified by phosphoproteomics were analyzed for protein‒protein interactions using the STRING protein interaction database. A protein‒protein interaction network was then constructed using Cytoscape v3.10.1.

### 2.15 Statistical analysis

All the data are presented as the mean ± SEM. Statistical analyses were carried out using GraphPad Prism software, employing one-way ANOVA for data analysis, with intergroup comparisons using Bonferroni correction. The cumulative survival rates were analyzed using Kaplan-Meier analysis combined with the log-rank test. A *p*-value of < 0.05 was considered to indicate statistical significance.

## 3 Results

### 3.1 AGNHW improved the 72-h survival rate and cerebral edema after LPS treatment

**FIGURE 1:**
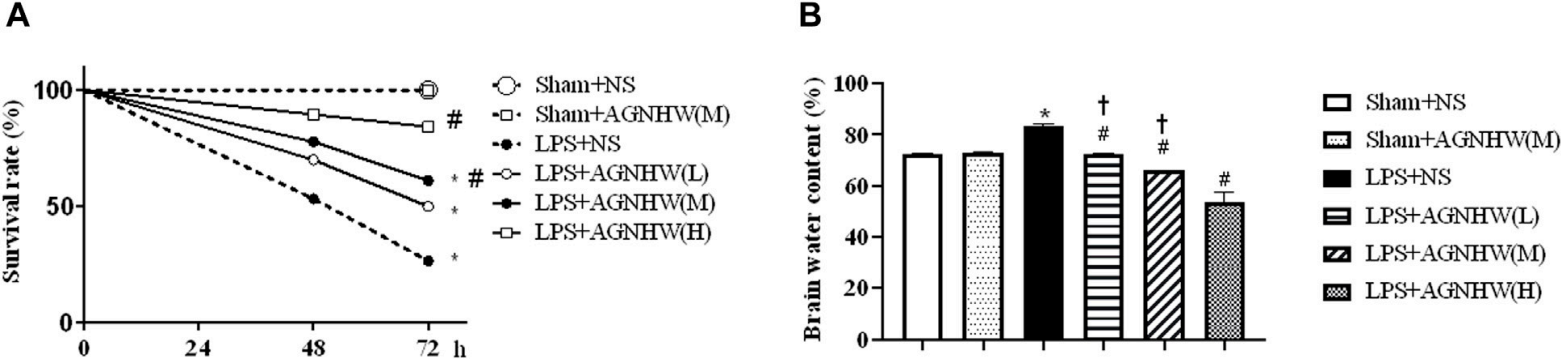
The effect of AGNHW on Survival Rates and Brain Water Content in Mice. **(A)** The 72-h survival rates of mice in each treatment group (N = 20). **(B)** Brain water content of the mice in each group (N = 6). The error bars represent the means ± SEMs. *p < 0.05 vs. Sham + NS, #*p* < 0.05 vs. LPS + NS, †*p* < 0.05 vs. LPS + AGNHW **(H)**.

We assessed the effect of AGNHW on the 72-h survival rate of mice after LPS treatment. Compared to that of the Sham + NS group, the survival rate of the LPS + NS group was significantly decreased (27%). Both medium and high doses of AGNHW (LPS + AGNHW(M) and LPS + AGNHW(H)) significantly mitigated this reduction in the 72-h survival rate (to 84% and 61%, respectively; Figure 1A).

Furthermore, mice treated with LPS exhibited significant cerebral edema. Compared to that in the Sham + NS group, the brain water content in the LPS + NS group was significantly increased. Low, medium, and high doses of AGNHW (LPS + AGNHW(L), LPS + AGNHW(M), and LPS + AGNHW(H)) all significantly inhibited the increase in the brain water content caused by LPS (Figure 1B).

### 3.2 AGNHW ameliorated LPS-induced cerebral microvascular permeability

**FIGURE 2:**
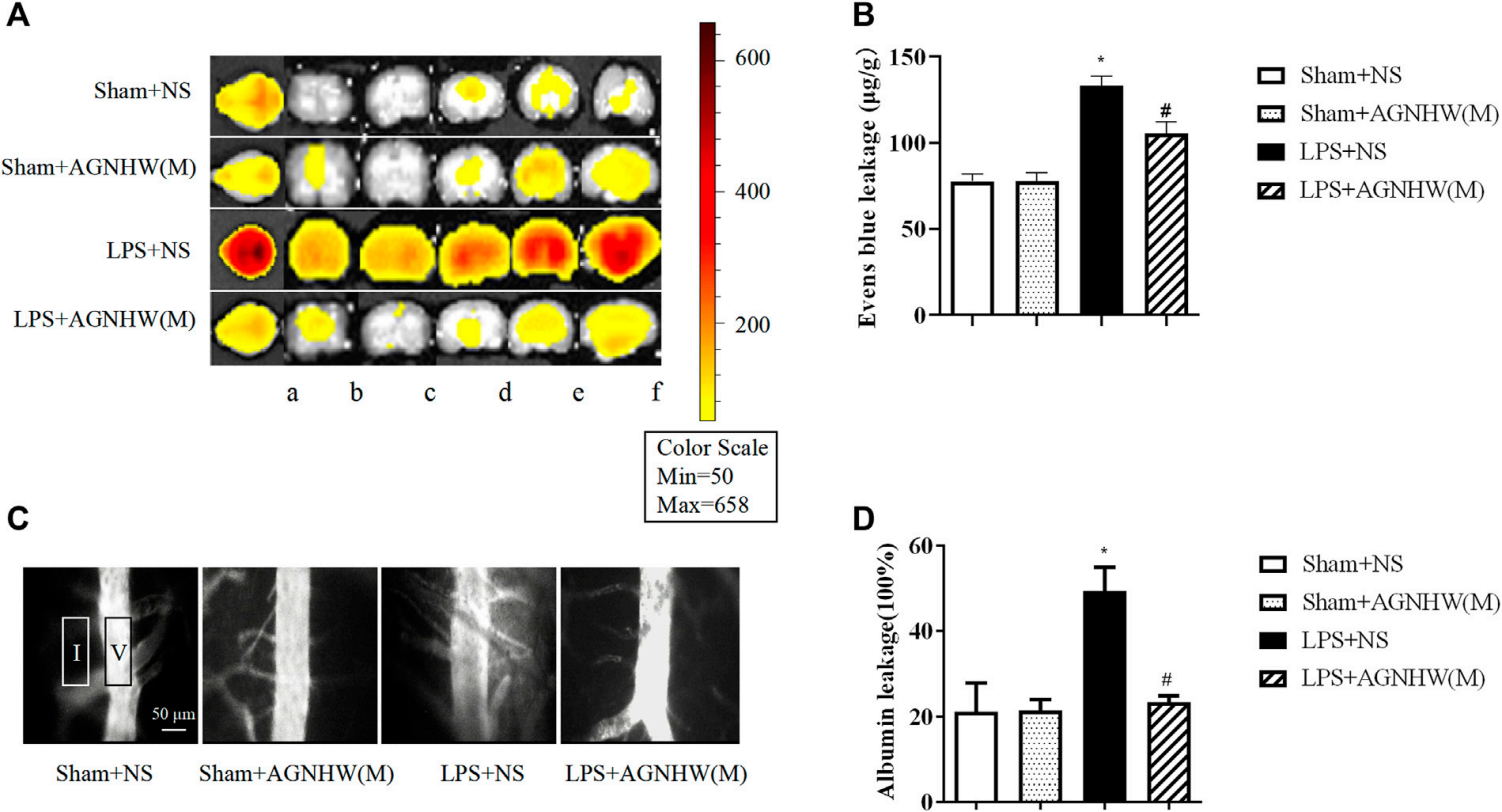
The Effect of AGNHW on Evans Blue Extravasation in Mouse Brain Tissue and FITC-Dextran Leakage in the Microvasculature on the Surface of the Mouse Brain. **(A)** Small-animal PET images of EB extravasation in mouse brain tissue. **(A)** Whole brain; b–f: brain tissue slices. **(B)** Results of spectrophotometry analysis of EB extravasation in mouse brain tissue (N = 6); the error bars represent the means ± SEMs. *p < 0.05 vs. Sham + NS, #*p* < 0.05 vs. LPS + NS. **(C)** Representative images of FITC-dextran leakage in mouse brain surface microvessels. **(D)** Statistical analysis of FITC-dextran leakage in mouse brain surface microvessels (N = 6); the error bars represent the means ± SEMs. *p < 0.05 vs. Sham + NS, #*p* < 0.05 vs. LPS + NS.

The brain tissue of mice injected with LPS or saline was imaged using small-animal PET at 48 h postinjection to assess Evans blue leakage, as shown in Figures 2A, B. Compared to that in the Sham + NS group, there was a noticeable increase in EB leakage in the brain tissue of the LPS + NS group. A medium dose of AGNHW (LPS + AGNHW(M)) significantly inhibited the LPS-induced leakage of Evans blue dye into brain tissue. Figure 2B shows the Evans blue content in the brain tissue homogenates as measured using a spectrophotometer (Figures 2A, B).

Additionally, fluorescence imaging and quantification of FITC-dextran leakage around the brain microvenules were performed 48 h after injection of LPS or saline. As shown in Figures 2C, D, the fluorescence intensity of FITC-dextran around the brain microvenules was significantly greater in the LPS + NS group than in the Sham + NS group. A medium dose of AGNHW (LPS + AGNHW(M)) significantly reduced the extravascular fluorescence intensity representing the leakage of FITC-dextran from cerebral microvenules. These results indicate that AGNHW significantly inhibited the increase in permeability of the cerebral microvasculature induced by LPS (Figures 2C, D).

### 3.3 AGNHW inhibited cell junction disruption induced by LPS

**FIGURE 3:**
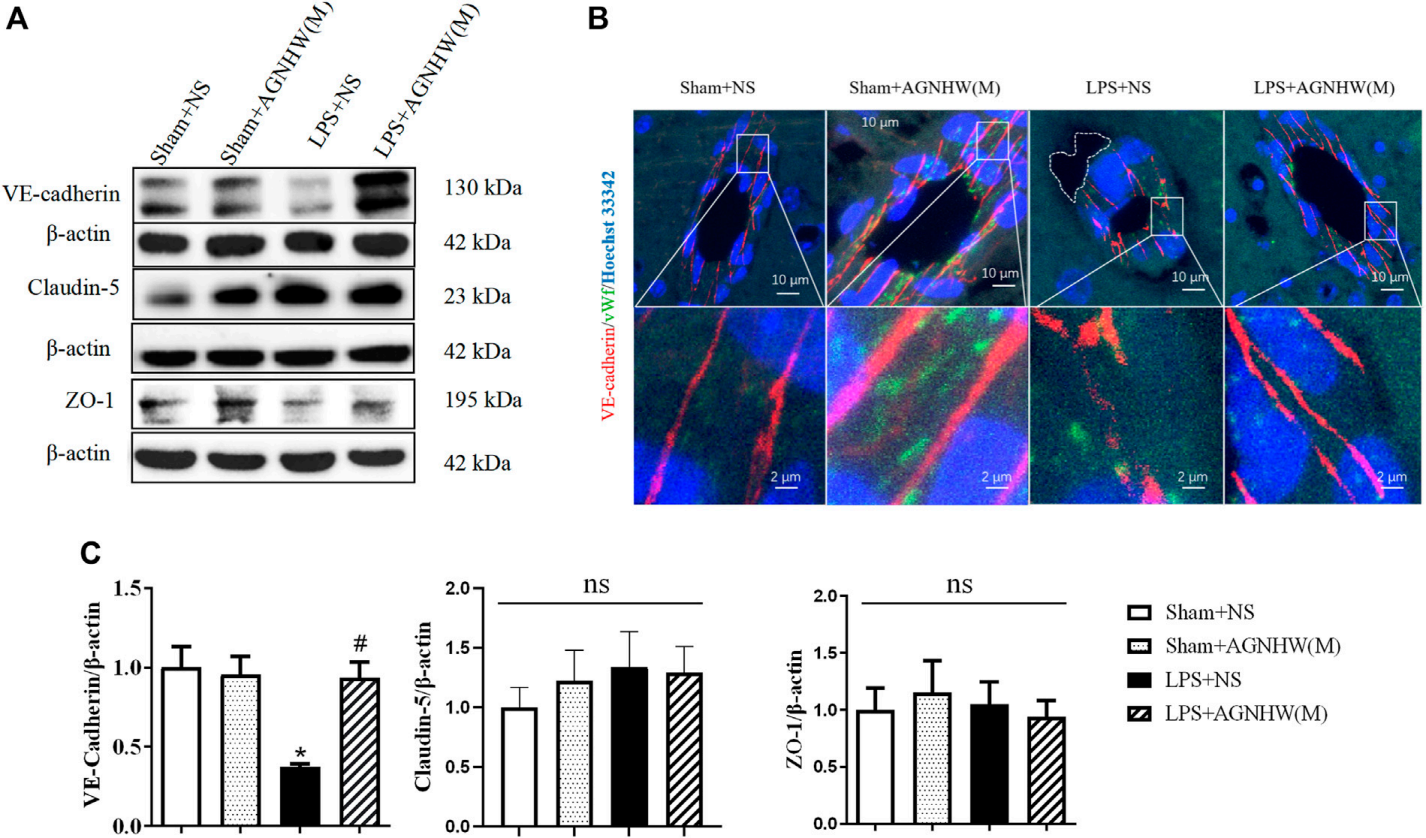
The effect of AGNHW on Cell-Cell Junction Protein Expression and Perivascular Edema in the Mouse Brain Microvasculature. **(A)** Western blot results showing the expression of cell‒cell junction proteins in mouse brain tissue. **(B)** Immunofluorescence staining images of frozen mouse brain tissue sections. VE-Cadherin in endothelial intercellular junctions is shown in red, the endothelial cell marker vWF is shown in green, and nuclei are shown in blue. The white dashed lines encircle perivascular edema in the brain endothelium (N = 5); the scale bar represents 10 μm. The area indicated by the white dashed line represents perivascular edema around cerebral microvessels. **(C)** Statistical analysis of the expression of cell‒cell junction proteins in the mouse brain (N = 6). The error bars represent the means ± SEMs. *p < 0.05 vs. Sham + NS, #*p* < 0.05 vs. LPS + NS.

The Western blot results for the intercellular junction proteins of vascular endothelial cells in brain tissue are presented in Figures 3A, C. The expression of the endothelial adhesion protein VE-Cadherin in the brain tissue microvascular endothelial cells in brain tissue was significantly lower in the LPS + NS group than in the Sham + NS group. A medium dose of AGNHW (LPS + AGNHW(M)) significantly inhibited the reduction in VE-Cadherin expression caused by LPS. There were no significant differences in the expression levels of the tight junction proteins Claudin-5 and ZO-1 among the groups (Figures 3A, C).

Figure 3B shows immunofluorescence images of VE-Cadherin (red) in brain tissue microvascular endothelial cells captured using a confocal microscope, with vWF (green) serving as an endothelial marker. In the brain tissue microvessels of the Sham + NS and Sham + AGNHW(M) groups, the distribution of VE-Cadherin was dense and continuous. In contrast, in the LPS + NS group, VE-Cadherin expression decreased, and there was noticeable edema around the brain tissue microvessels (indicated by the white dashed lines). A medium dose of AGNHW (LPS + AGNHW(M)) markedly inhibited the disruption and reduction in VE-Cadherin induced by LPS, thereby alleviating edema around brain tissue microvessels (Figure 3B).

**FIGURE 4:**
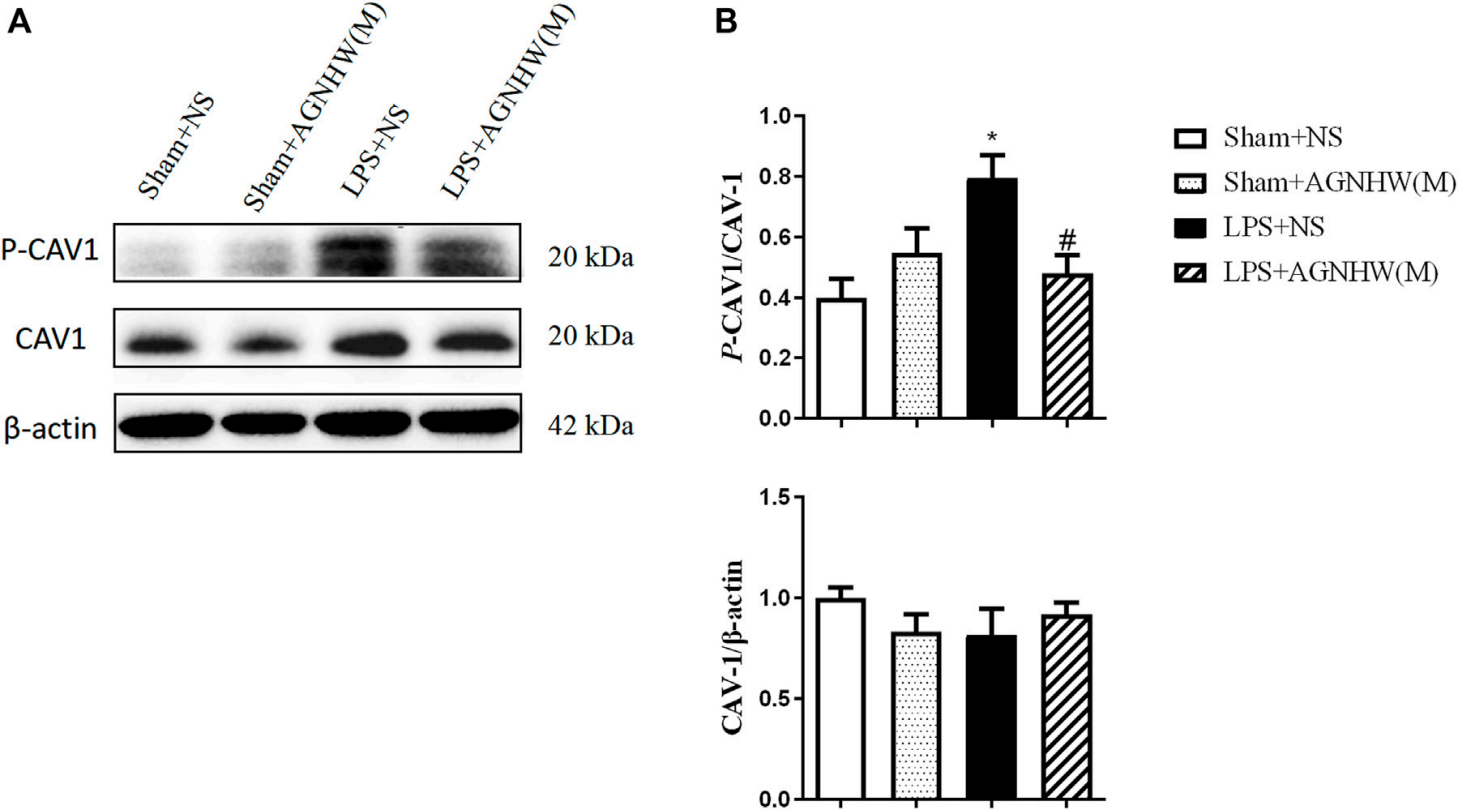
The Effect of AGNHW on the Phosphorylation of CAV-1 in Mouse Brain Tissue **(A)** Western blot results showing the expression of CAV-1 and phosphorylation of CAV-1 in the mouse brain. **(B)** Statistical graph of the expression of CAV-1 and phosphorylation of CAV-1 in the mouse brain (N = 6). The error bars represent the means ± SEMs. *p < 0.05 vs. Sham + NS, #*p* < 0.05 vs. LPS + NS.

The Western blot results for the expression and phosphorylation of CAV-1 are shown in Figures 4A, B. Compared to those in the Sham + NS group, the phosphorylation levels of CAV-1 in the endothelial cells of the LPS + NS group were significantly elevated. A medium dose of AGNHW (LPS + AGNHW(M)) significantly inhibited the phosphorylation of CAV-1 induced by LPS. There were no significant changes in the protein expression of CAV-1 among the groups (Figures 4A, B).

### 3.4 AGNHW promoted the membrane expression of AQP4 in LPS-Treated mouse brain tissue

**FIGURE 5:**
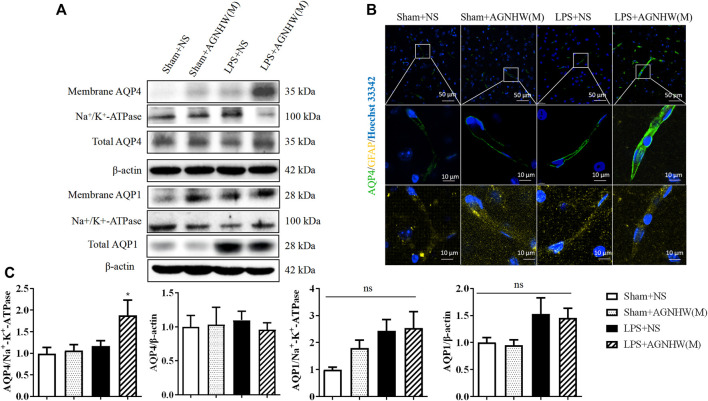
The effect of AGNHW on the Total and Membrane Expression of AQP4 and AQP1 in Mouse Brain Tissue **(A)** Western blot results showing the expression of AQP1 and AQP4 in the cell membrane and total AQP1 and AQP4 in the mouse brain. **(B)** Immunofluorescence staining images of frozen mouse brain tissue sections. AQP4 is shown in green, GFAP in yellow, and nuclei in blue (N = 5). The scale bar represents 10 μm. **(C)** Statistical analysis of AQP1 and AQP4 expression in the cell membrane and total AQP1 and AQP4 expression in mouse brain tissue by Western blot (N = 6). The error bars represent the means ± SEMs. *p < 0.05 vs. Sham + NS.

Figures 5A, C show the Western blot results for the expression of AQP1 and AQP4 in the membranes and cytoplasm of cells. There were no significant changes in the membrane expression or total protein expression of AQP4 in the brain tissue of the LPS + NS group compared to the Sham + NS group. The total protein expression of AQP4 in the LPS + AGNHW(M) group also did not change significantly; however, LPS + AGNHW(M) significantly upregulated the membrane expression of AQP4 in astrocytes in mouse brain tissue. Following LPS stimulation, the protein expression of another water channel protein, AQP1, did not significantly change compared to that in the Sham + NS group; similarly, the membrane expression of AQP1 did not significantly increase in the LPS + NS group. AGNHW did not have a noticeable effect on the protein or membrane expression of AQP1 (Figures 5A, C).

Figure 5B shows the immunofluorescence staining images of astrocytic AQP4 (green), which were captured with a confocal microscope, with GFAP (yellow) as an astrocyte marker. Compared to that in the Sham + NS group, there was no significant difference in AQP4-positive staining in the LPS + NS group. However, compared to the LPS + NS group, the LPS + AGNHW(M) group exhibited a noticeable increase in AQP4-positive staining in astrocytes, with the expression of AQP4 was primarily localized on the cell membranes (Figure 5B).

### 3.5 Temporal phosphoproteomic analysis identified differences in protein phosphorylation status

**FIGURE 6:**
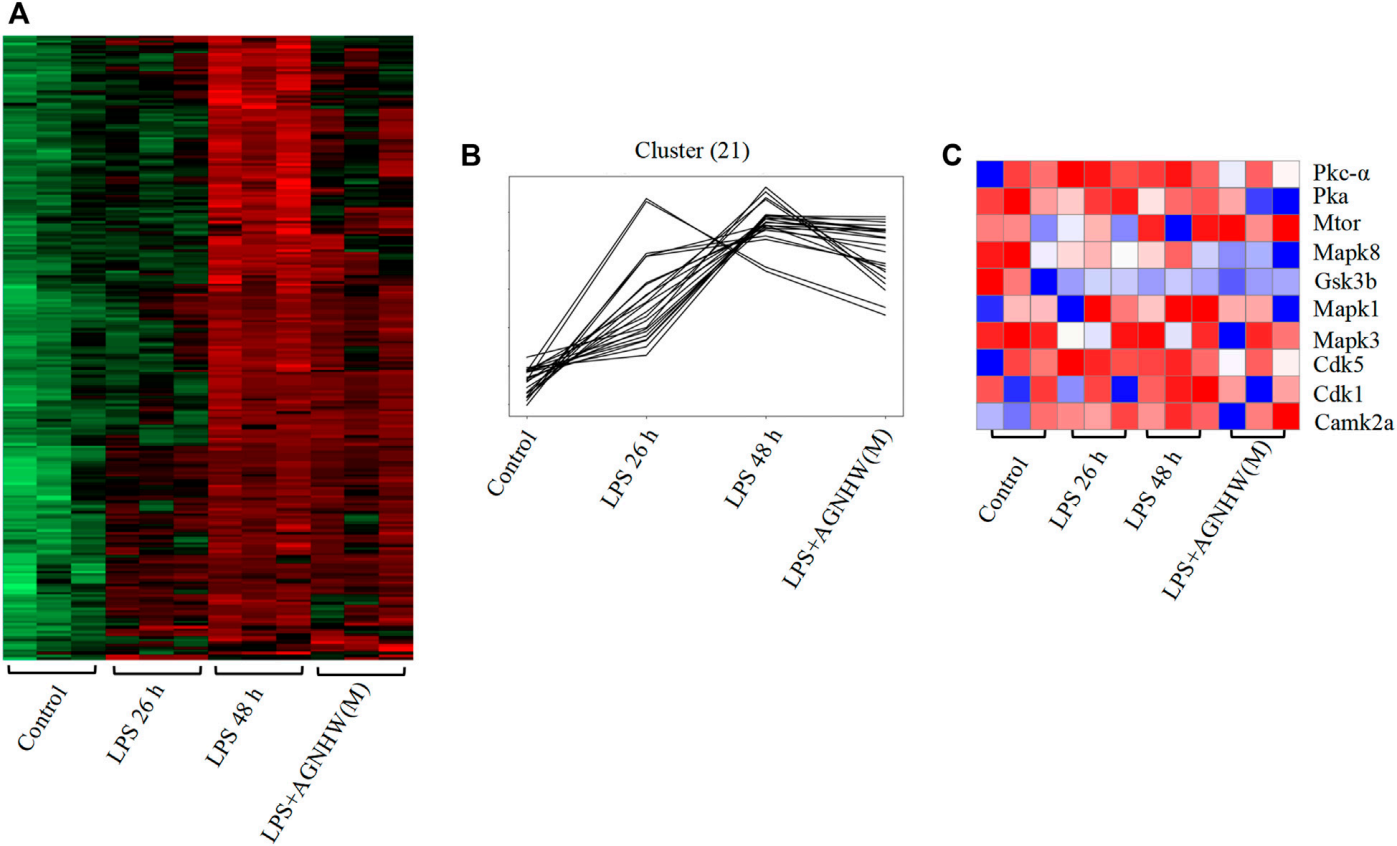
Differential Phosphoproteomic Analysis **(A)** Heatmap of the differential expression of phosphorylated proteins in mouse brain tissue; *p* < 0.05, fold change ≥1.5. **(B)** Line graph showing increased levels of phosphorylated proteins after LPS stimulation compared to 0 h and decreased levels of phosphorylated proteins after AGNHW treatment. **(C)** Heatmap of PTM-SEA kinase activity analysis results.

Phosphoproteomic analysis of brain tissues at 0 h (control), 26 h (LPS, 26 h), and 48 h (LPS, 48 h and LPS + AGNHW (M)) was performed to assess protein phosphorylation status. Proteins exhibiting phosphorylation fold-change values greater than or equal to 1.5 (*p* < 0.05) were selected, and a total of 240 proteins were identified. Among these proteins, 21 exhibited increased phosphorylation in the LPS group compared to the 0 h group and decreased phosphorylation after AGNHW treatment (Figures 6A, B). PTM-SEA kinase activity analysis was used to analyze the kinase activity, and 10 key kinases were identified (Figure 6C). Finally, analysis of the protein‒protein interaction network between the targets of AGNHW blood metabolites and the phosphoproteome revealed key proteins that may be regulated by the blood metabolites of AGNHW. The activities of PKC-α and MAPK1, which can downregulate the expression of VE-Cadherin, were decreased after AGNHW administration. PKC-α can also phosphorylate CAV-1. The two key kinases regulating the membrane translocation of AQP4 that exhibited changes in activity were Protein kinase A (PKA) and PKC-α. PKC-α activity increased after LPS stimulation and decreased after AGNHW administration. PKA activity did not increase after LPS stimulation but decreased after AGNHW administration (Figure 6C).

**FIGURE 7:**
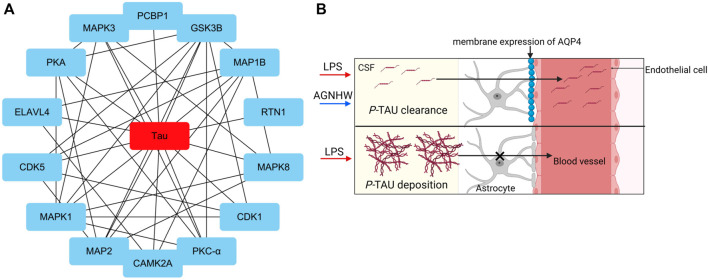
Core Proteins of the Phosphoprotein Interaction Network **(A)** Diagram of the core network of protein‒protein interactions, with P-Tau at the center. **(B)** Schematic representation of *P*-Tau as a marker for cerebral edema clearance, where P-Tau does not accumulate in brain tissue when AQP4 has normal edema clearance capabilities and accumulates when AQP4 has impaired edema clearance capabilities.

Analysis of the protein‒protein interactions among the factors identified by phosphoproteomics revealed that phosphorylated Tau (*P*-Tau) is the core protein in this network. Compared to that in the control group, the expression of *P*-Tau was upregulated following LPS injection, and AGNHW inhibited the LPS-induced upregulation of *P*-Tau expression (Figure 7A). *P*-Tau is a marker of the brain’s fluid clearance capacity, and an increase in its level indicates an impairment in the ability to clear cerebral edema (Figure 7B).

**FIGURE 8:**
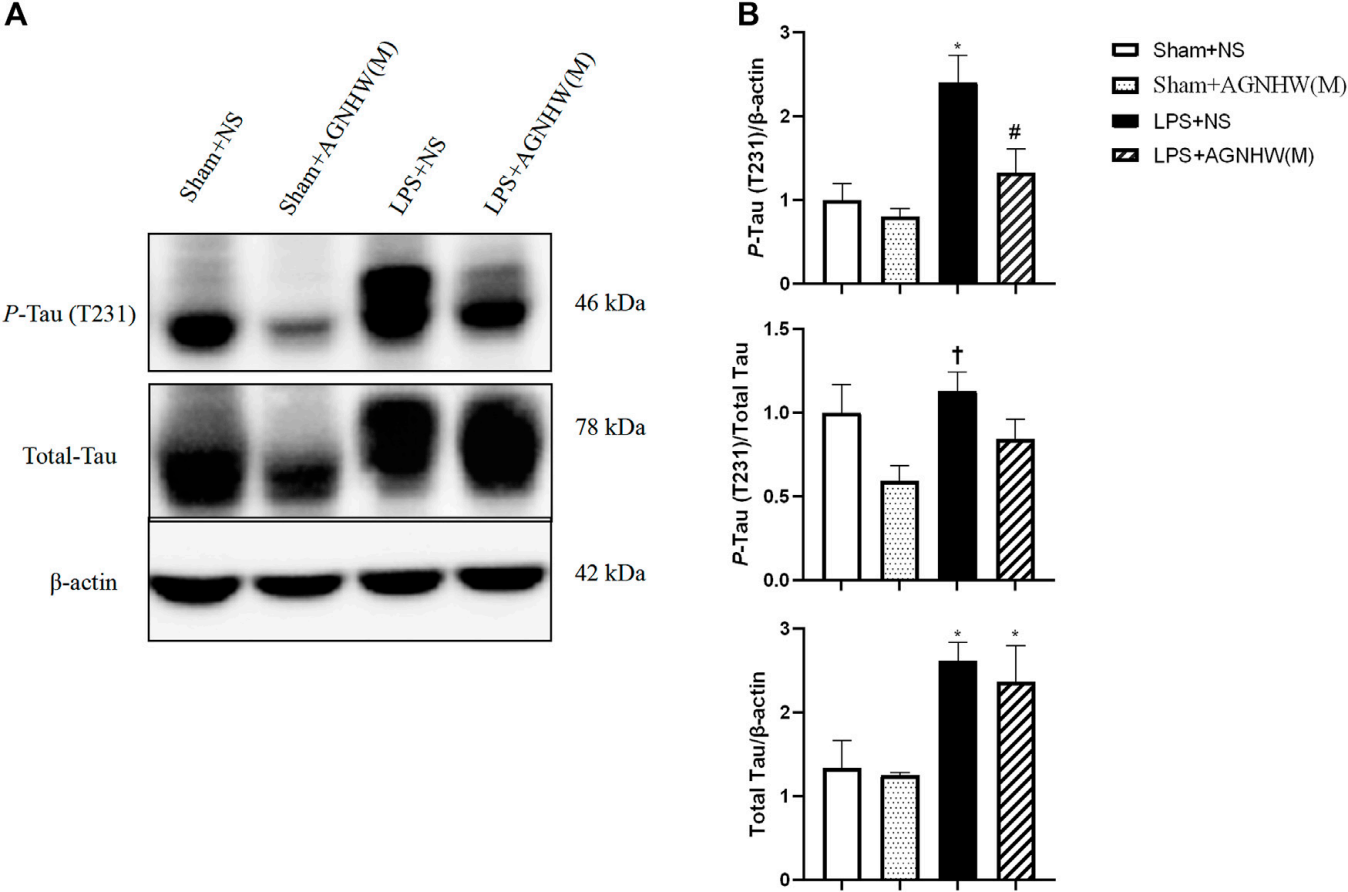
Effects of AGNHW on P-Tau Expression, Tau Phosphorylation Levels, and Total Tau Expression **(A)** Western blot results showing the expression of Tau and the phosphorylation of Tau in the mouse brain. **(B)** Statistical graph of the expression of Tau and the phosphorylation of Tau in the mouse brain (N = 6). The error bars represent the means ± SEMs. *p < 0.05 vs. Sham + NS, #*p* < 0.05 vs. LPS + NS, †*p* < 0.05 vs. Sham + AGNHW (M).

We used Western blotting to verify the effects of LPS stimulation and AGNHW administration on P-Tau. Figures 8A, B show the Western blot results for phosphorylated Tau protein (T231) and total Tau protein expression levels. Compared to the Sham + NS group, the LPS group exhibited increased expression of P-Tau in brain tissue, while AGNHW inhibited this increase. In terms of the regulation of Tau phosphorylation levels, Tau phosphorylation was not significantly increased in the LPS + NS group compared to that in the Sham + NS group. Similarly, AGNHW administration did not significantly inhibit the increase in Tau phosphorylation compared to that in the LPS + NS group. Compared to that in the Sham + AGNHW (M) group, the level of phosphorylated Tau was significantly greater in the LPS group. Finally, total Tau expression was significantly increased in the LPS + NS group compared to the Sham + NS group, and AGNHW administration did not reduce the expression of total Tau (Figures 8A, B).

### 3.6 Detection of AGNHW metabolites in the blood

**FIGURE 9:**
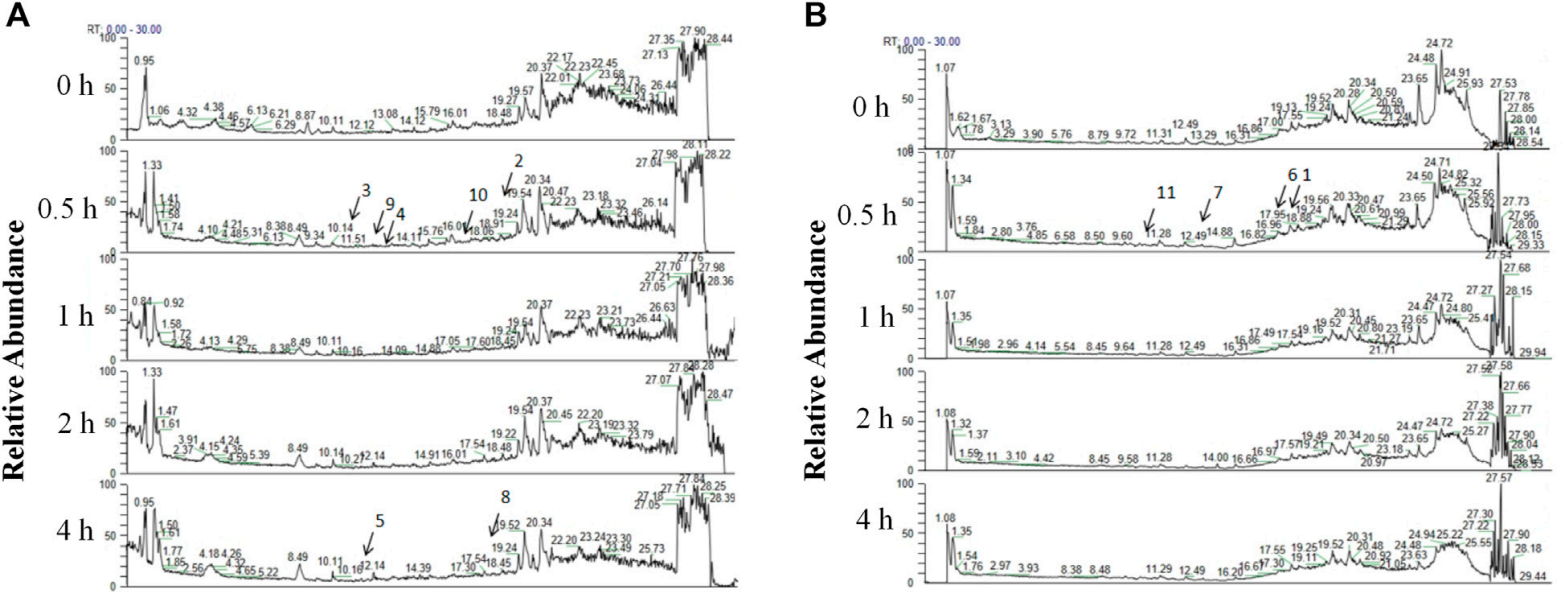
Analysis of AGNHW Metabolites in the Bloodstream **(A)** Total ion flow of mouse plasma in the positive ion mode for retention times of 0–30 min. The arrows indicate the AGNHW metabolites detected in the bloodstream. 2: Alphitolic acid (APA); 3: 4,10-epizedoarondiol (EDD); 4: germacr-1 (10)-ene-5,8-dione (GED); 5: octanoic acid (OA); 8: 7Z-tetradecenoic acid (TA); 9: bisabola-1,3,5,7 (14),10-pentaene (BP); 10: (1E,6E, 8R)-1-methyl-5-methylene-8-prop-1-en-2-ylcyclodeca-1,6-dien (MMPEYD). **(B)** Total ion flow of mouse plasma in negative ion mode for retention times of 0–30 min. The arrows indicate the metabolites of AGNHW detected in the bloodstream. 1: Asiatic acid (ASA); 6: gardenolic acid B (GAB); 7: curcolonol (CCN); 11: 4-(1-Hydroxyvinyl)phenol (HP).

A total of 11 prototype metabolites or AGNHW metabolites were identified in the blood using the aforementioned methods. Prototype metabolites included 4,10-epizedoarondiol (EDD), germacr-1 (10)-ene-5,8-dione (GED) and curcolonol (CCN) from *Curcumae Radix*; asiatic acid (ASA) and alphitolic acid (APA) from *Borneolum Syntheticum*; and gardenolic acid B (GAB) from *Gardeniae Fructus*. Two kinds of gut bacterial metabolites were identified, including octanoic acid (OA), which is produced by the metabolism of methyl hexanoate in *Scutellariae Radix*, and 7Z-tetradecenoic acid (TA), which is produced by the metabolism of palmitic acid in *Curcumae Radix*. Additionally, three kinds of liver CYP450 enzyme metabolites were identified: (1E,6E, 8R)-1-methyl-5-methylene-8-prop-1-en-2-ylcyclodeca-1,6-dien (MMPEYD), which is produced by the metabolism of Germacrene D from *Scutellariae Radix*; 4-(1-hydroxyvinyl)phenol (HP), which is produced by the metabolism of acetophenone; and bisabola-1,3,5,7 (14), 10-pentaene (BP), which is produced by the metabolism of curcumol from *Curcumae* Radix (Figure 9A, B; Supplementary S1A-K; Supplementary Table S2).

### 3.7 Phosphoproteomic analysis of AGNHW blood metabolite interaction with targets and differentially expressed proteins

**FIGURE 10:**
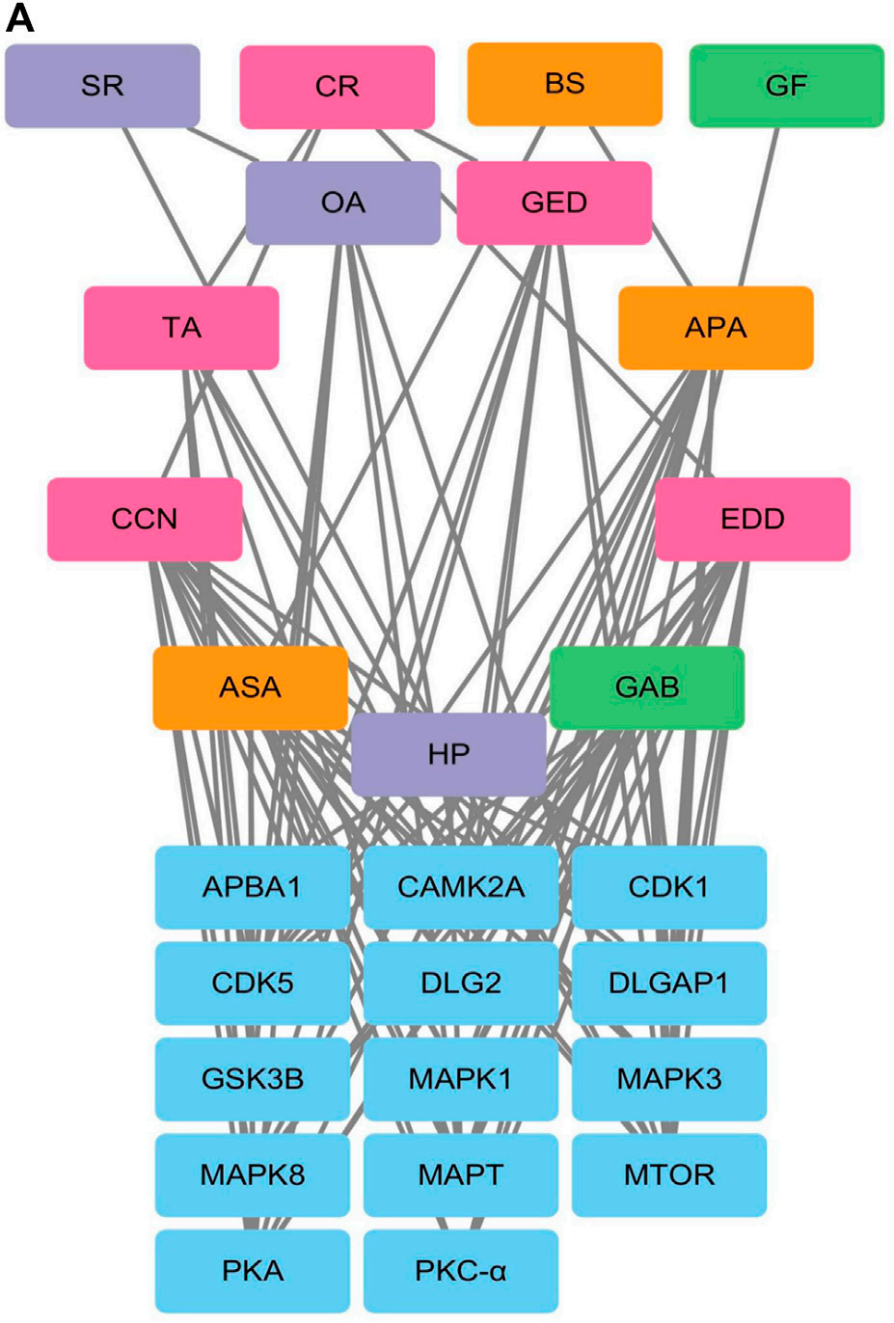
Regulatory Network between Differentially Phosphorylated Proteins and AGNHW Blood Metabolites **(A)** Protein‒protein interaction network between blood metabolites of AGNHW and differentially phosphorylated proteins according to phosphoproteome data. SR: *Scutellariae Radix*; CR: *Curcumae Radix*; BS: *Borneolum Syntheticum*; GF: *Gardeniae Fructus.*

Phosphoproteomic analysis of the interaction network between the blood metabolites of AGNHW and the differentially expressed proteins revealed that PKC-α can disassemble VE-Cadherin, activate CAV, and regulate AQP4. Since AGNHW can reduce the activation of PKC-α and MAPK1 caused by LPS, we focused on blood metabolites that can regulate PKC-α and MAPK1. Specifically, the AGNHW blood metabolites ASA, CCN, APA, EDD, and GAB may be associated with PKC-α and MAPK1 (Figure 10A).

### 3.8 Effects of seven AGNHW blood metabolites on AQP4 and PKC-ɑ in astrocyte

**FIGURE 11:**
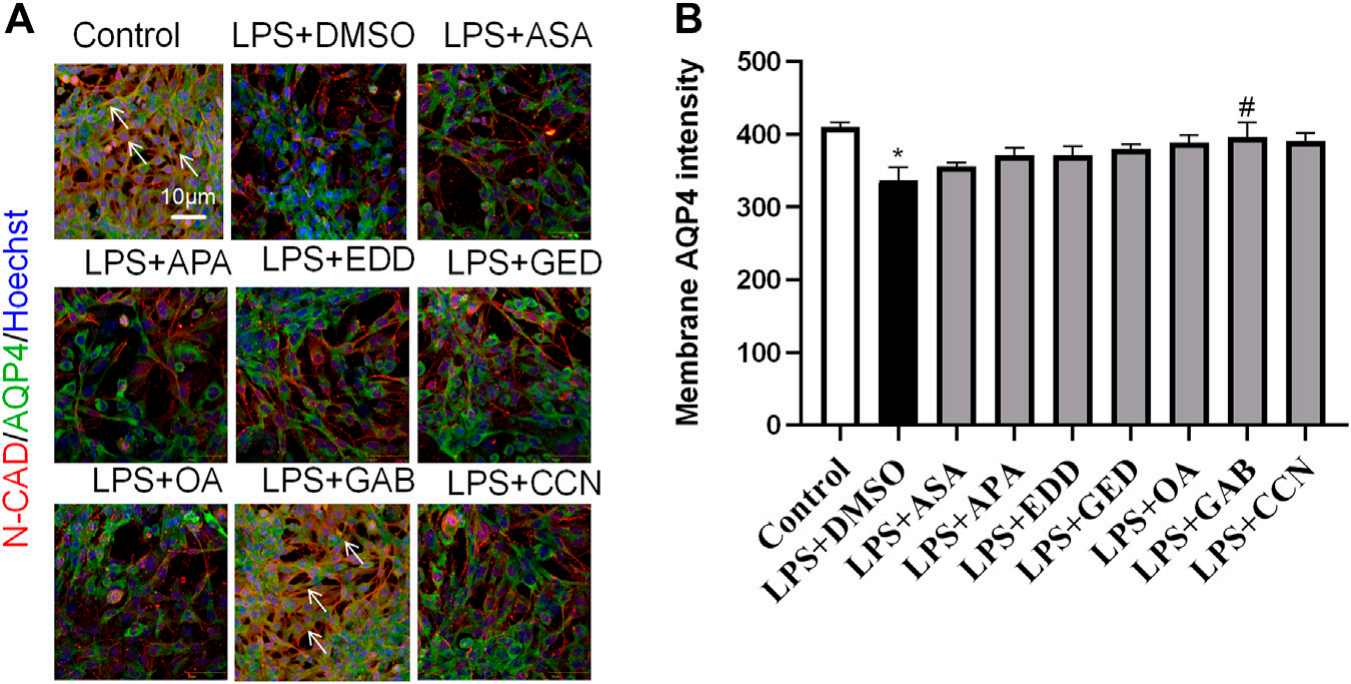
High-Content Screening Results of the Blood-Brain Barrier Cell Model **(A)** Immunofluorescence images of the blood‒brain barrier cell model. N-Cadherin in astrocyte membranes is shown in red, AQP4 is shown in green, and nuclei are shown in blue. The white arrows indicate AQP4 on astrocyte membranes. (N = 6); the scale bar represents 10 μm. **(B)** Statistical analysis of AQP4 expression on astrocyte membranes. The error bars represent the means ± SEMs. *p < 0.05 vs. Sham + NS, #*p* < 0.05 vs. LPS + NS.

**FIGURE 12:**
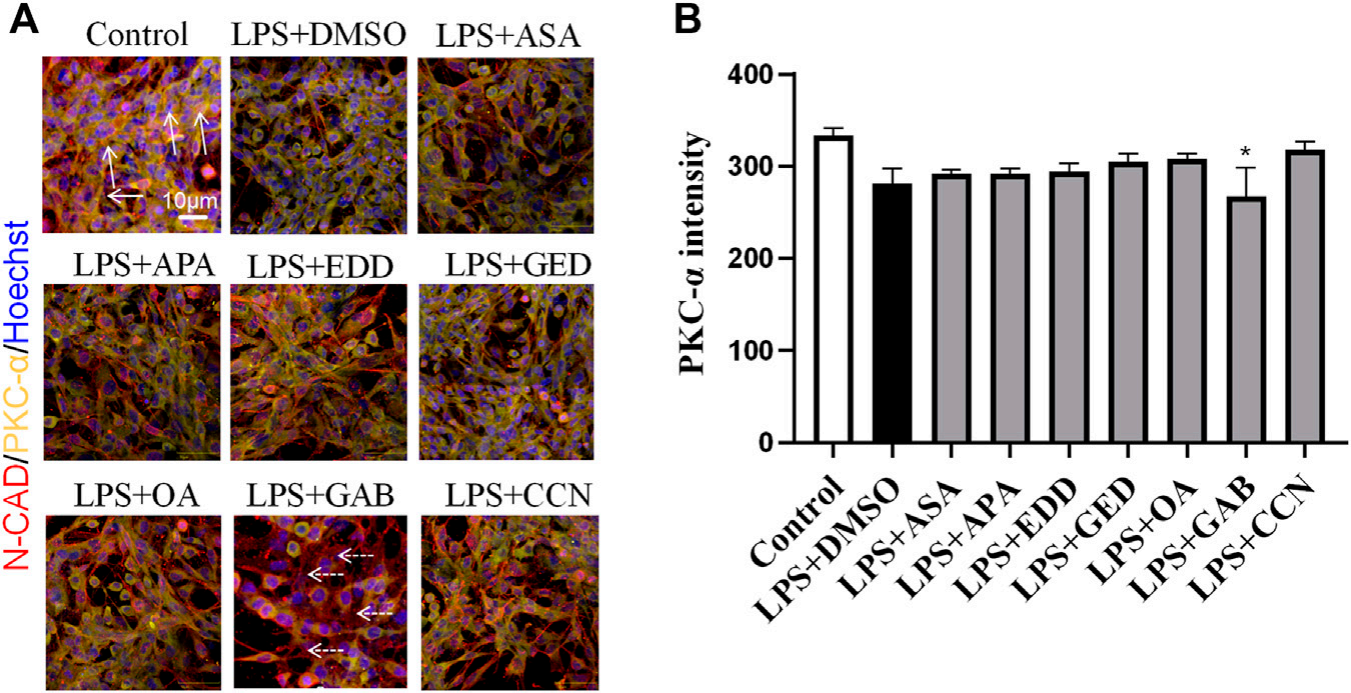
High-Content Screening Results of the Blood-Brain Barrier Cell Model **(A)** Immunofluorescence images of the blood‒brain barrier cell model. N-Cadherin in astrocyte membranes is shown in red, PKC-ɑ is shown in yellow, and nuclei are shown in blue. The white arrows indicate PKC-ɑ. The white dashed arrows indicate the region with reduced PKC-α(N = 6); the scale bar represents 10 μm. **(B)** Statistical analysis of PKC-α expression on astrocyte The error bars represent the means ± SEMs. *p < 0.05 vs. Sham + NS.

To explore the effects of AGNHW blood metabolites on LPS-induced AQP4 and PKC-α expression in astrocyte, we applied LPS (1 μg/mL) to a blood‒brain barrier cell model at 0 and 24 h. At the 26th hour, seven AGNHW blood metabolites were added separately to the culture medium at 10^−5^ mol/L. Immunofluorescence staining was performed 48 h later, and the average fluorescence intensity of AQP4 at single-pixel points of N-Cadherin (N-CAD) was recorded using a microlens dual spinning disk confocal high-content system. AQP4 expression on the astrocyte membrane was significantly reduced in the LPS + DMSO group, significantly increased in the LPS + GAB group, and showed no significant differences in the LPS + ASA, LPS + APA, LPS + EDD, LPS + GED, LPS + OA, and LPS + CCN groups compared to that in the control group (Figures 11A, B). Additionally, The expression of PKC-ɑ was significantly reduced in the LPS + GAB group compared to the control group, while no significant differences were observed in the other groups (Figures 12A, B).

## 4 Discussion

This study confirmed that AGNHW could ameliorate the reduction in survival rate and the cerebral edema caused by LPS in mice. AGNHW also inhibited LPS-induced leakage of FITC-dextran and Evans blue into the brain. AGNHW suppressed the low expression of VE-Cadherin in cerebral microvascular endothelial cells, inhibited the phosphorylation of CAV-1 and promoted the membrane expression of AQP4.

Sepsis-induced cerebral edema is a high-mortality disease for which there are no known effective drugs (Perner et al., 2012). AGNHW is a well-known classic prescription for treating infection-induced unconsciousness. However, whether AGNHW can also improve vascular-origin brain edema caused by endotoxin is unclear. This study established a vascular-origin cerebral edema mouse model through two intraperitoneal injections of LPS at 0 and 24 h. Using this model, we demonstrated that the administration of AGNHW 2 h after the second LPS injection reduced mortality, inhibited FITC-dextran and Evans blue leakage, and alleviated cerebral edema. These results provide reliable evidence for the treatment of sepsis-induced vascular-origin cerebral edema with AGNHW.

The primary pathways for cerebral microvascular leakage are the paracellular pathway, which is mediated by endothelial cell junctions, and the transcellular pathway, which is mediated by caveolae (Pan et al., 2015). Using immunofluorescence staining and Western blot analyses, this study demonstrated that AGNHW can ameliorate both the reduced expression of VE-Cadherin and the phosphorylation of CAV-1 in cerebral tissue induced by LPS, indicating that AGNHW limits both paracellular and transcellular leakage. These findings suggested that AGNHW can improve vascular-origin cerebral edema caused by LPS by inhibiting cerebral microvascular permeability.

The key finding of this study was that AGNHW could promote the membrane expression of AQP4 in cerebral tissue. AQP4 plays opposing roles in vasogenic cerebral edema and cytotoxic cerebral edema. For example, ischemic stroke mainly manifests as cytotoxic cerebral edema, during which AQP4 exacerbates cellular swelling (Zador et al., 2009). In contrast, LPS primarily causes blood-brain barrier disruption, leading to vasogenic cerebral edema (Peng et al., 2021). Previous studies have confirmed the crucial role of AQP4 in astrocytes in facilitating the reabsorption of fluid that has leaked into surrounding areas during the occurrence of vasogenic cerebral edema (Zhang et al., 2022). We showed that LPS did not change the expression or localization of AQP4 in astrocytes. However, AGNHW enhanced the levels of membrane-localized AQP4 in astrocytes, suggesting an enhanced ability to reabsorb edema around cerebral microvessels (Papadopoulos et al., 2004). Thus, the effect of AGNHW in eliminating cerebral edema may be mediated by increased AQP4 membrane expression.

Furthermore, phosphoproteomic analysis showed that PKA activity was increased at 26 h and decreased at 48 h after LPS injection. PKC-α activity was also increased and was inhibited by AGNHW. Previous studies have shown that PKA promotes the membrane translocation of AQP4, while PKC inhibits this process (Moeller et al., 2009). Our study showed that AGNHW inhibited PKC-α activity but did not affect PKA activity, suggesting that AGNHW promotes AQP4 membrane translocation by inhibiting PKC-α activity.

This study also identified 11 AGNHW metabolites that entered the bloodstream. Among these, the prototype metabolites of AGNHW include EDD, GED, and CCN from Curcumae Radix; GAB from Gardeniae Fructus; ASA and APA from Borneolum Syntheticum; and metabolites metabolized by intestinal bacteria, including OA, which is derived from methyl cinnamate, a prototype metabolite of Scutellariae Radix; and TA, which is derived from palmitic acid, a prototype metabolite of Curcumae Radix. Additionally, three metabolites metabolized by liver CYP450 enzymes were detected, including BP derived from turmerone (in Curcumae Radix), MMPEYD derived from gimeracil D (in Scutellariae Radix), and HP derived from acetophenone (in Scutellariae Radix). The provision of these metabolites in the blood may be a key contribution to the effects of AGNHW. Through reverse targeting of metabolites and phosphoproteomic analysis of differential protein‒protein interactions, we found that ASA, CCN, APA, EDD, and GAB could regulate PKC-α to inhibit the low expression of VE-Cadherin caused by LPS and inhibit the phosphorylation of CAV-1. These compounds can also regulate MAPK1 to inhibit the LPS-induced decrease in VE-Cadherin expression. Through *in vitro* cell culture experiments, we found that GAB can promote the membrane expression of AQP4 in astrocytes, while simultaneously reducing the expression of PKC-ɑ in astrocytes. These results suggest that the improvement of LPS-induced vascular-origin cerebral edema in mice by AGNHW involves the joint action of its main metabolites.

## 5 Conclusion

This study confirmed that AGNHW can inhibit LPS-induced vasogenic cerebral edema in mice. This effect is associated with the inhibition of the LPS-induced decrease in VE-Cadherin expression in mouse brain tissue and inhibition of CAV-1 phosphorylation and upregulation of AQP4 expression on astrocyte membranes in the mouse brain after LPS injection. Our research provides new insights into the mechanisms by which AGNHW can treat vascular-origin edema caused by LPS.

## Data Availability

The datasets presented in this study can be found in ProteomeXchange with identifier PXD054069; available at https://proteomecentral.proteomexchange.org/ui?search=PXD054069.

## Abbreviations

AGNHW, AnGongNiuHuangWan; AQP1, Aquaporin 1; AQP4, Aquaporin 4; CAV-1, Caveolin-1; FITC, Fluorescein isothiocyanate; LPS, lipopolysaccharides; MAPK1, Mitogen-activated protein kinase 1; MCA, Middle cerebral artery; P-CAV-1, P-Caveolin-1; PKA, Protein kinase A; PKC-α, Protein kinase C-α; N-CAD, N-Cadherin; PTM-SEA, Posttranslational Modification Signature Enrichment Analysis; Ti-IMAC, Ti-immobilized Metal Affinity Chromatography; VE-Cadherin, Vascular endothelial Cadherin; ZO-1, Zonula Occludens-1.
