# Supplementary material for: Angong Niuhuang Wan ameliorates LPS-induced cerebrovascular edema by inhibiting blood‒brain barrier leakage and promoting the membrane expression of AQP4
Source: Front Pharmacol. 2024 Aug 1;15:1421635. doi: 10.3389/fphar.2024.1421635 (PMC11324430; doi:10.3389/fphar.2024.1421635)
Supplement: Supplementary file 2 [file Table1.docx]

Supplemental Table 1 Characterization of the Botanical drugs Included in AGNHW

| Botanical drugs | Percentage content (%) |
| --- | --- |
| *Coptidis Rhizoma* | 10 |
| *Gardeniae Fructus* | 10 |
| *Scutellariae Radix* | 10 |
| *Curcumae Radix* | 10 |
| *Artificialis Calculus Bovis* | 10 |
| *Artificialis Moschus* | 2.5 |
| *Cinnabaris* | 10 |
| *Borneolum Syntheticum* | 2.5 |
| *Bubali Cornu* | 20 |
| *Margarita* | 5 |
| *Realgar* | 10 |

**Supplemental Table 2 (Related to Figure 8). Identification of metabolites of AGNHW absorbed into mice blood by HPLC/Q-TOF-MS.**

|  | **Source** | **Identification** | **Molecular formula** | **Measured m/z** | **Calculated m/z** | **RT/min** | **IonModel** |
| --- | --- | --- | --- | --- | --- | --- | --- |
| **1** | ***Borneolum Syntheticum*** | **Asiatic acid (ASA)** | **C30H48O5** | **487.34273** | **487.3429** | **18.53** | **[M-H]-1** |
| **2** | ***Borneolum Syntheticum*** | **Alphitolic acid (APA)** | **C30H48O4** | **473.36261** | **473.36254** | **19.07** | **[M-H]-1** |
| **3** | ***Scutellariae Radix*** | **Octanoic acid (OA)** | **C8H16O2** | **145.12243** | **145.12231** | **11.27** | **[M+H]+1** |
| **4** | ***Scutellariae Radix*** | **(1E,6E,8R)-1-methyl-5-methylidene-8-prop-1-en-2-ylcyclodeca-1,6-diene (MMPEYD)** | **C15H22** | **203.1794** | **203.17943** | **16.48** | **[M+H]+1** |
| **5** | ***Scutellariae Radix*** | **4-(1-Hydroxyvinyl)phenol (HP)** | **C8H8O2** | **135.04471** | **135.04515** | **11.17** | **[M-H]-1** |
| **6** | ***Curcumae Radix*** | **4,10-Epizedoarondiol (EDD)** | **C15H24O3** | **253.17983** | **253.17982** | **14.33** | **[M+H]+1** |
| **7** | ***Curcumae Radix*** | **Germacr-1(10)-ene-5,8-dione (GED)** | **C15H24O2** | **237.18501** | **237.1849** | **12.02** | **[M+H]+1** |
| **8** | ***Curcumae Radix*** | **Curcolonol (CCN)** | **C15H20O4** | **263.12893** | **263.12888** | **15.93** | **[M-H]-1** |
| **9** | ***Curcumae Radix*** | **7Z-tetradecenoic acid (TA)** | **C14H26O2** | **227.20054** | **227.20056** | **18.21** | **[M+H]+1** |
| **10** | ***Curcumae Radix*** | **Bisabola-1,3,5,7(14),10-pentaene (BP)** | **C15H20** | **201.16381** | **201.16378** | **13.69** | **[M+H]+1** |
| **11** | ***Gardeniae Fructus*** | **Gardenolic acid B (GAB)** | **C30H46O5** | **485.32722** | **485.32725** | **17.38** | **[M-H]-1** |

**
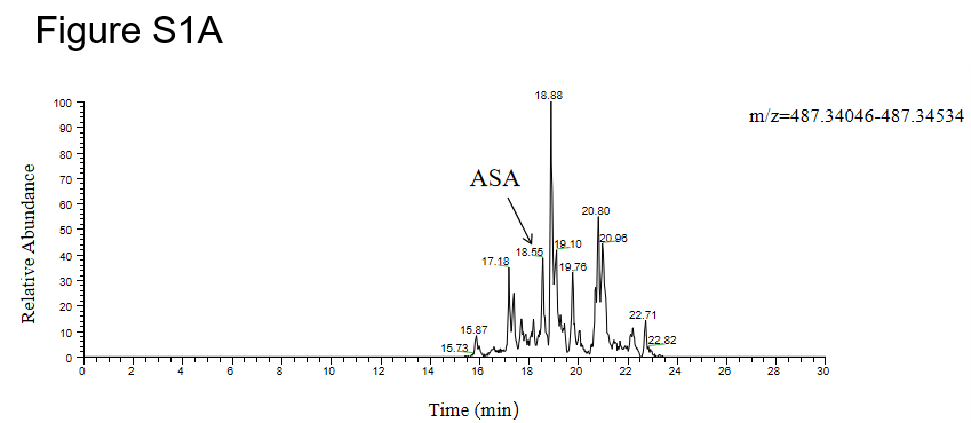
**

**Supplemental Figure S1A(Related to Figure 8)** Chromatogram of ASA

**
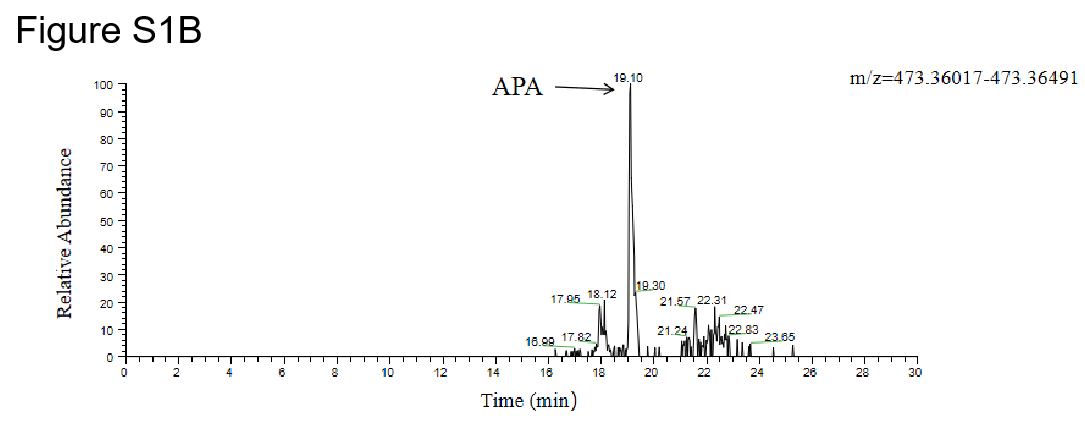
**

**Supplemental Figure S1B(Related to Figure 8)** Chromatogram of APA

**
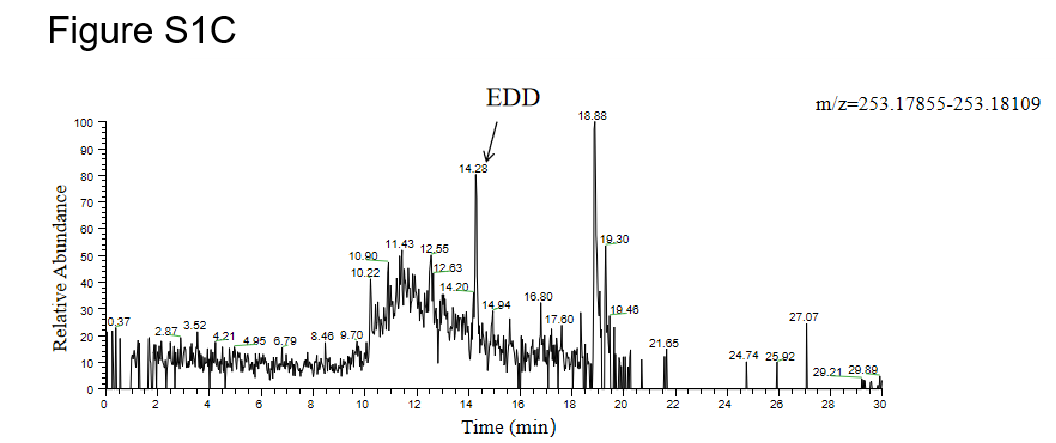
**

**Supplemental Figure S1C(Related to Figure 8)** Chromatogram of EDD


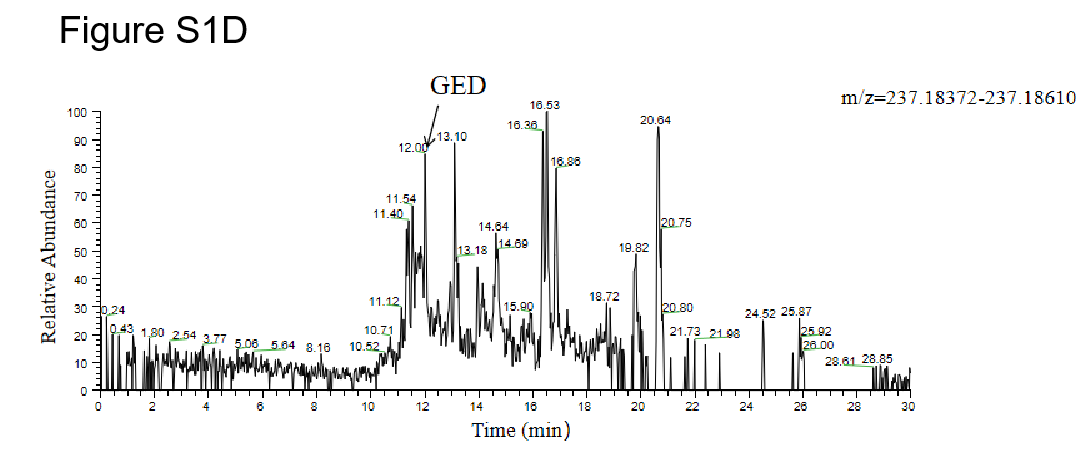


**Supplemental Figure S1D(Related to Figure 8)** Chromatogram of GED

**
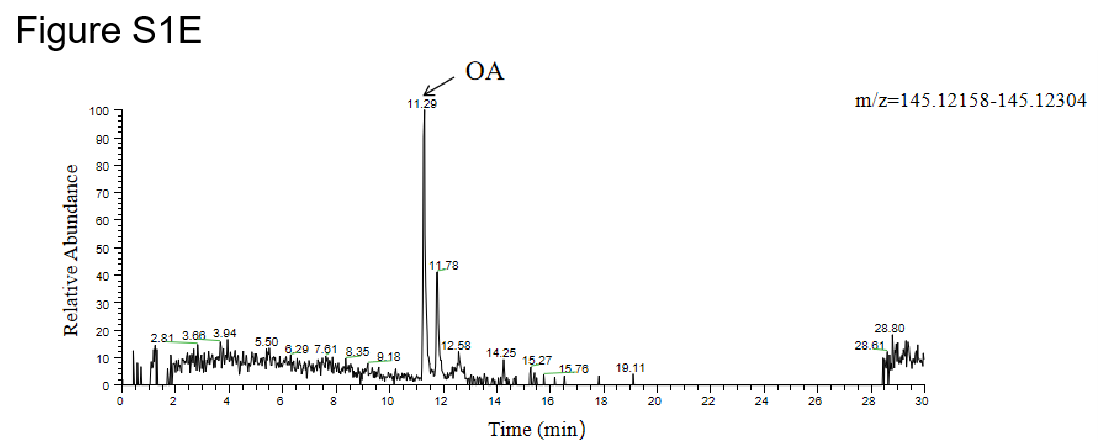
**

**Supplemental Figure S1E(Related to Figure 8)** Chromatogram of OA

**
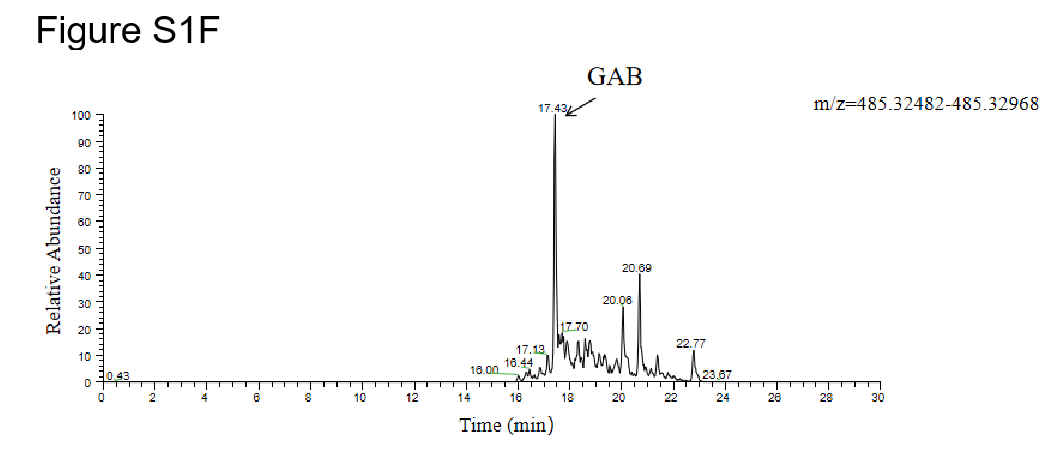
**

**Supplemental Figure S1F(Related to Figure 8)** Chromatogram of GAB

**
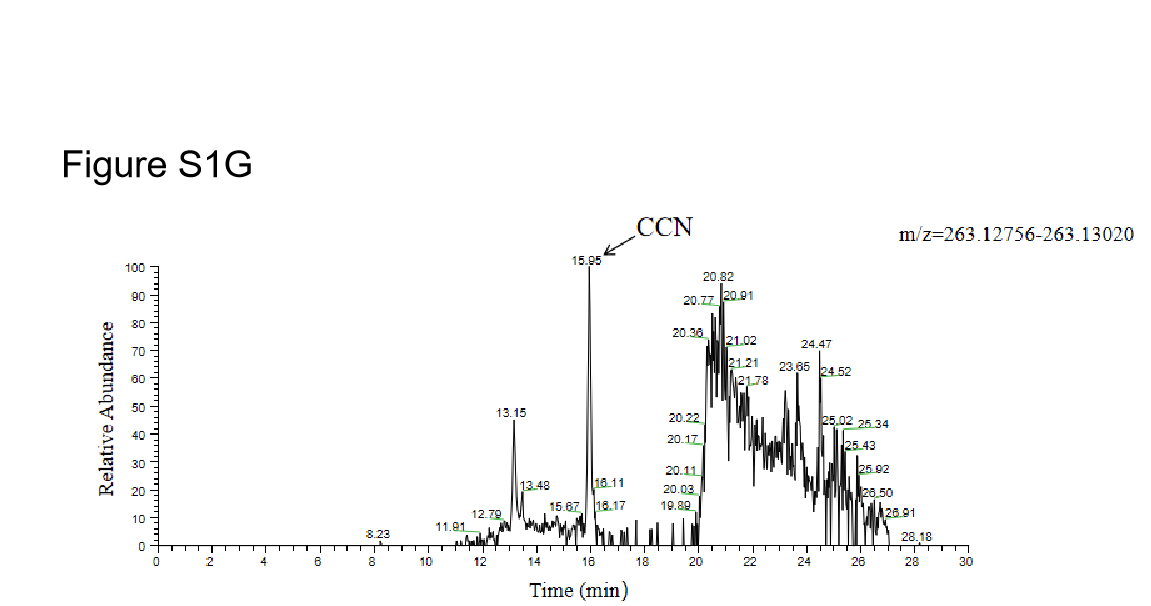
**

**Supplemental Figure S1G(Related to Figure 8)** Chromatogram of CCN

**
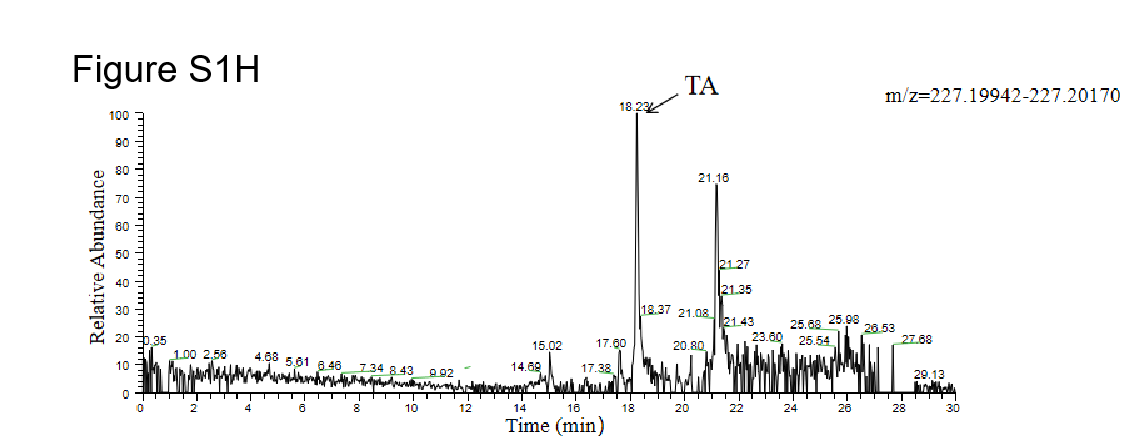
**

**Supplemental Figure S1H(Related to Figure 8)** Chromatogram of TA

**
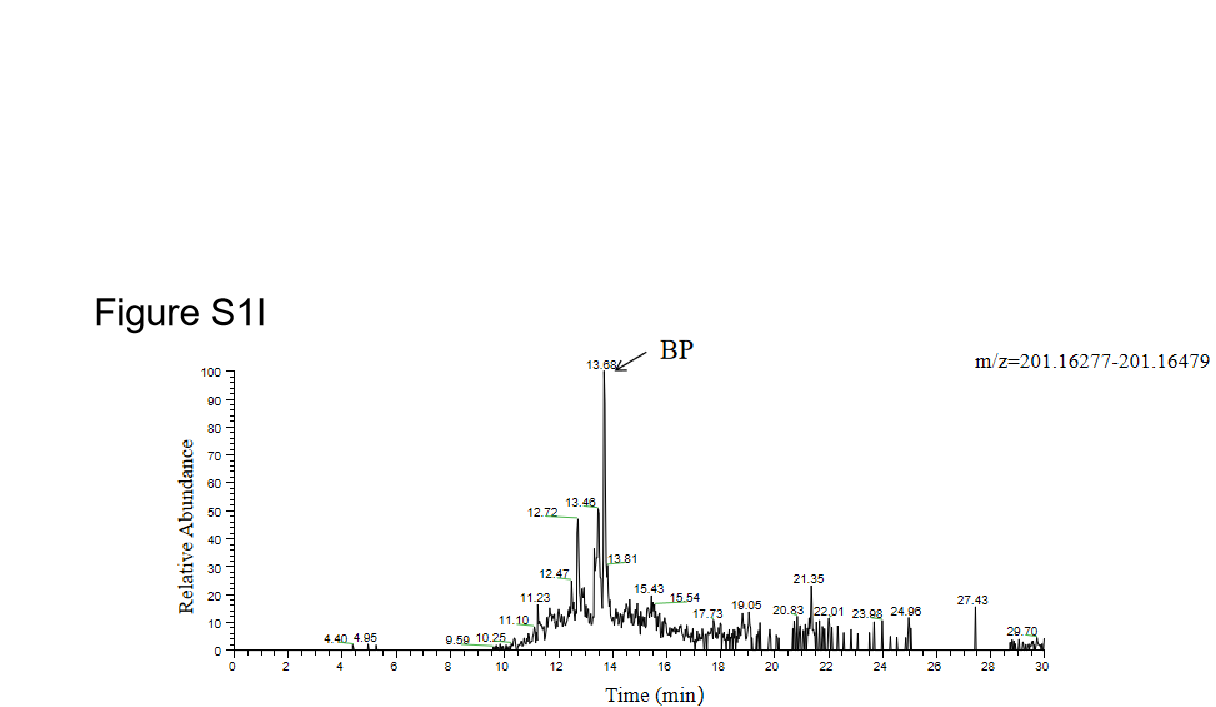
**

**Supplemental Figure S1I(Related to Figure 8)** Chromatogram of BP

**
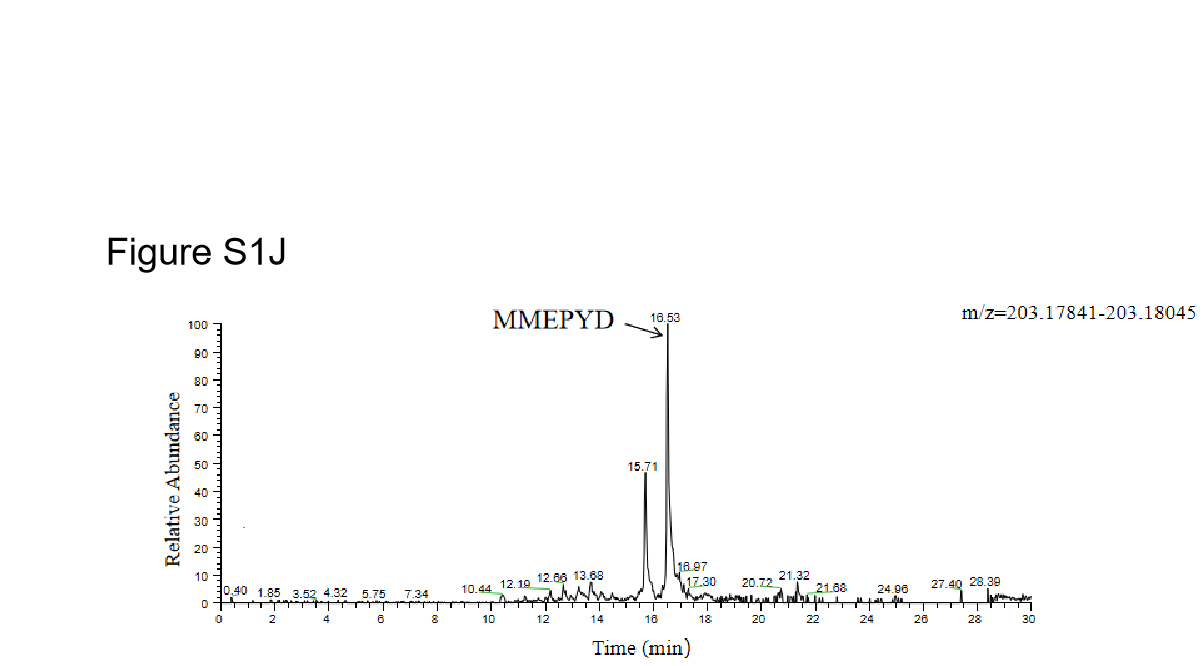
**

**Supplemental Figure S1J(Related to Figure 8)** Chromatogram of MMEPYD

**
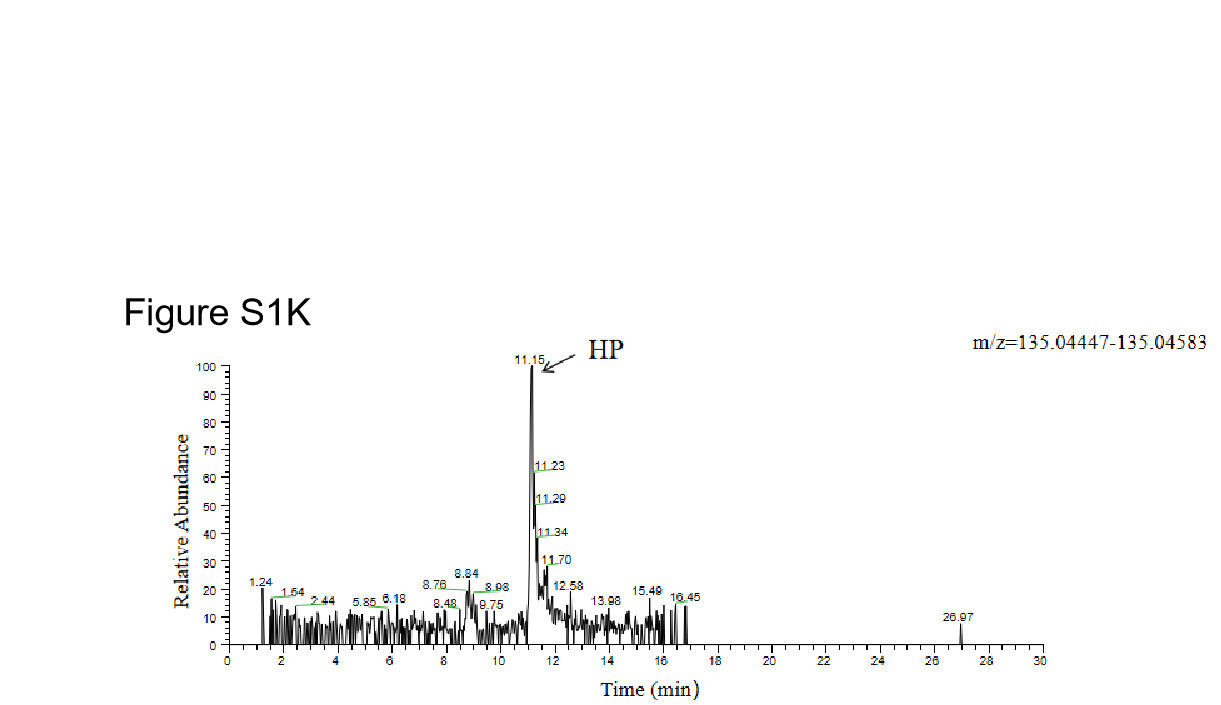
**

**Supplemental Figure S1K(Related to Figure 8)** Chromatogram of HP
