# Supplementary material for: Angong Niuhuang Wan ameliorates LPS-induced cerebrovascular edema by inhibiting blood‒brain barrier leakage and promoting the membrane expression of AQP4
Source: Front Pharmacol. 2024 Aug 1;15:1421635. doi: 10.3389/fphar.2024.1421635 (PMC11324430; doi:10.3389/fphar.2024.1421635)
Supplement: Supplementary file 3 [file DataSheet3.PDF]

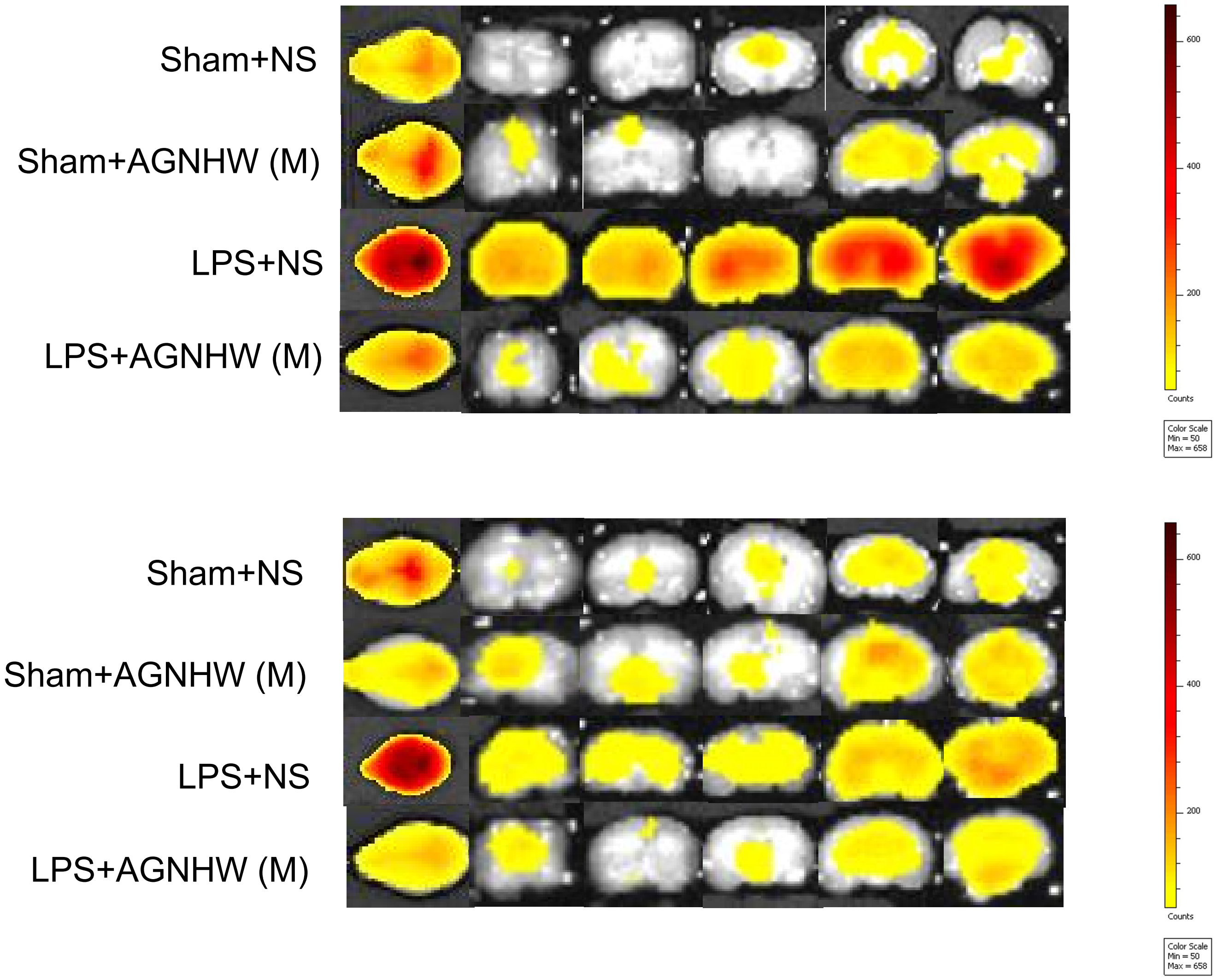

Sham+NS
Sham+AGNHW (M)
LPS+NS
LPS+AGNHW (M)
600
400
200
Counts
Color Scale
Min = 50
Max = 658
Sham+NS
Sham+AGNHW (M)
LPS+NS
LPS+AGNHW (M)
600
400
200
Counts
Color Scale
Min = 50
Max = 658

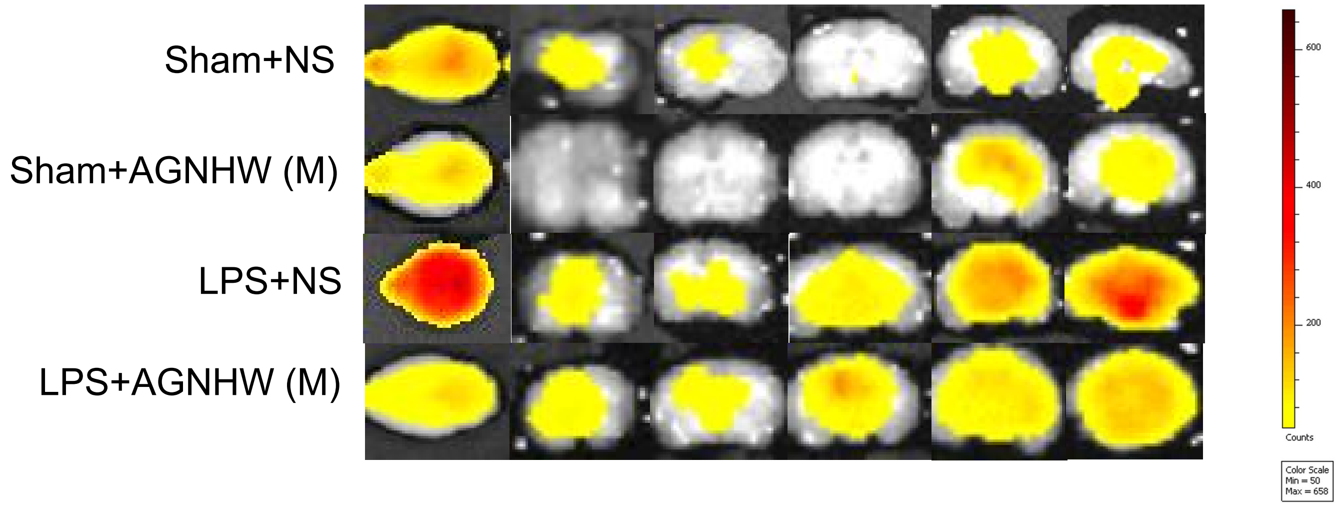
Sham+NS
Sham+AGNHW (M)
LPS+NS
LPS+AGNHW (M)
600
400
200
Counts
Color Scale
Min = 50
Max = 658

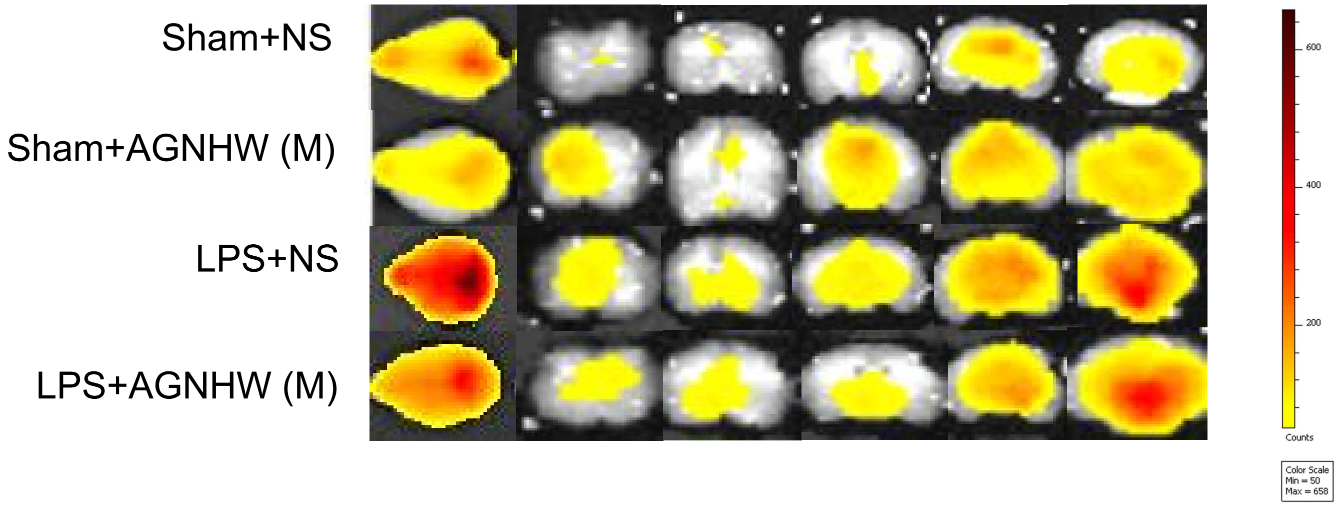
Sham+NS
Sham+AGNHW (M)
LPS+NS
LPS+AGNHW (M)
600
400
200
Counts
Color Scale
Min = 50
Max = 658

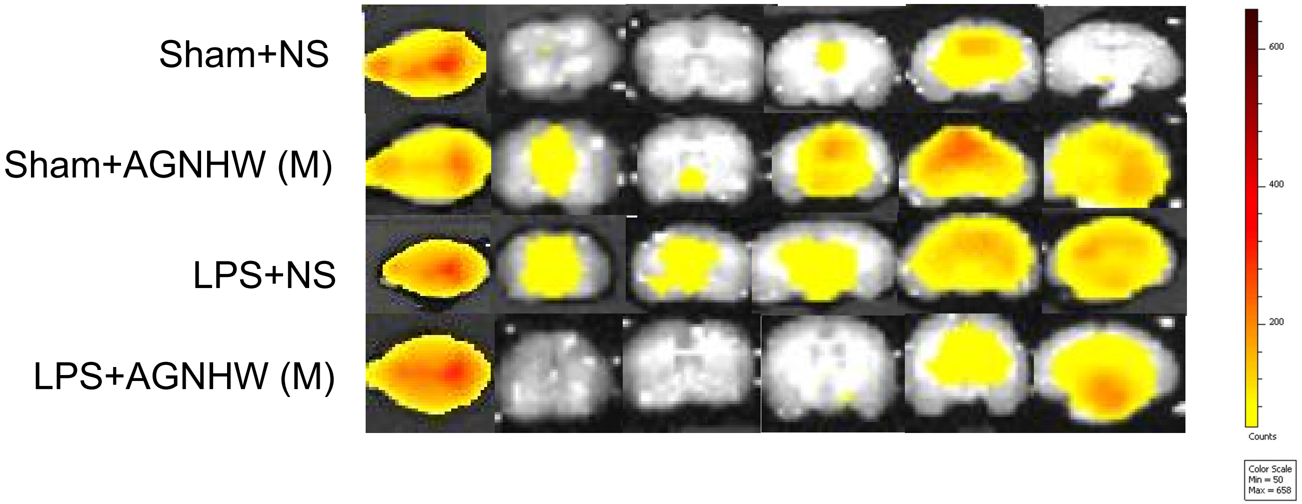

Sham+NS
Sham+AGNHW (M)
LPS+NS
LPS+AGNHW (M)
600
400
200
Counts
Color Scale
Min = 50
Max = 658

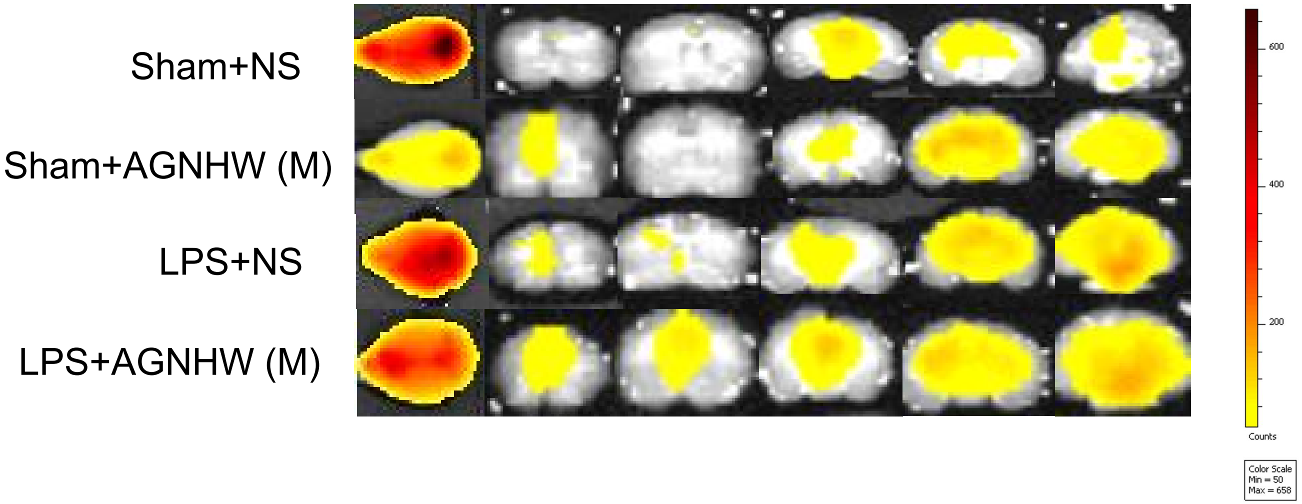

Sham+NS
Sham+AGNHW (M)
LPS+NS
LPS+AGNHW (M)
600
400
200
Counts
Color Scale
Min = 50
Max = 658

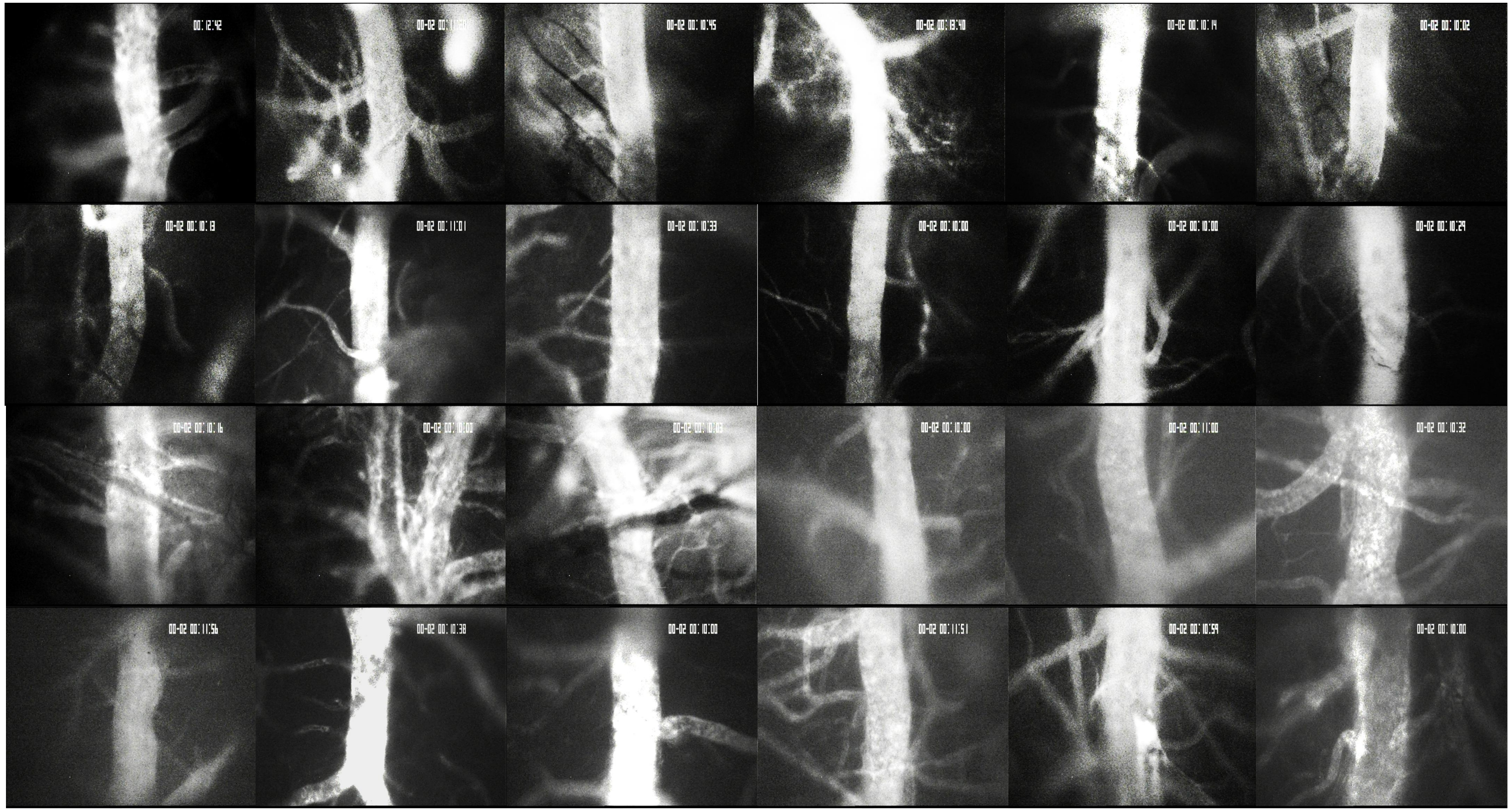

Sham+NS

Sham+AGNHW (M)

LPS+NS

LPS+AGNHW (M)

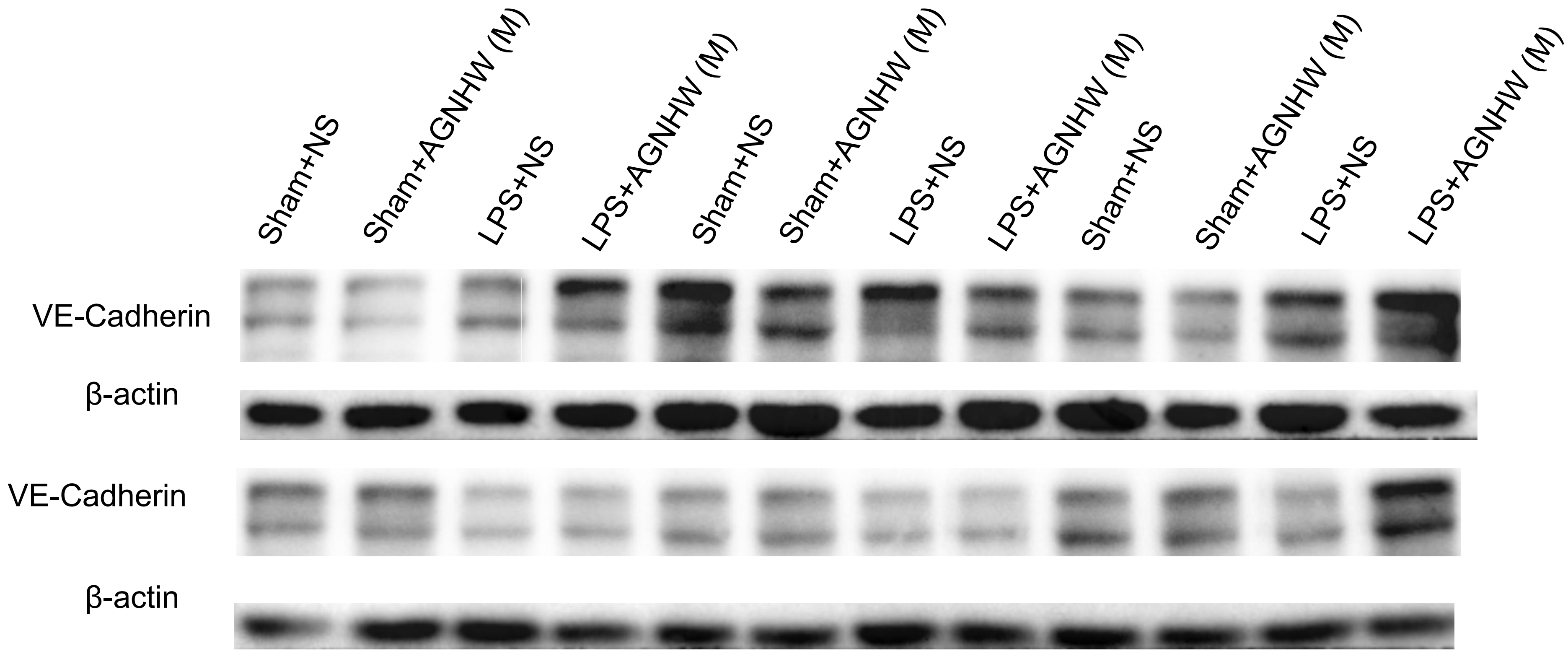
Sham+NS
Sham+AGNHW (M)
LPS+NS
LPS+AGNHW (M)
Sham+NS
Sham+AGNHW (M)
LPS+NS
LPS+AGNHW (M)
Sham+NS
Sham+AGNHW (M)
LPS+NS
LPS+AGNHW (M)
VE-Cadherin
β-actin
VE-Cadherin
β-actin

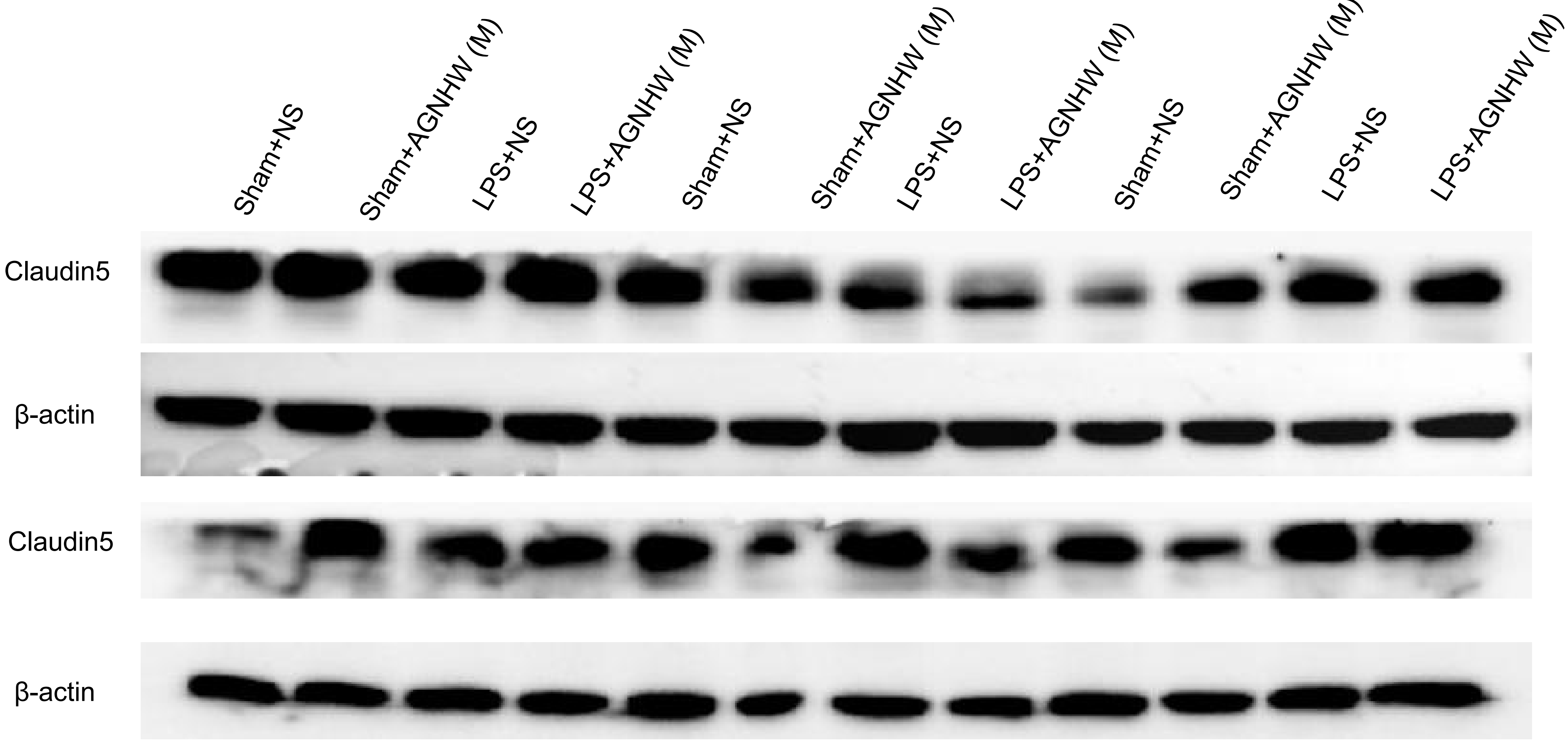
Sham+NS
Sham+AGNHW (M)
LPS+NS
LPS+AGNHW (M)
Sham+NS
Sham+AGNHW (M)
LPS+NS
LPS+AGNHW (M)
Sham+NS
Sham+AGNHW (M)
LPS+NS
LPS+AGNHW (M)
Claudin5
β-actin
Claudin5
β-actin

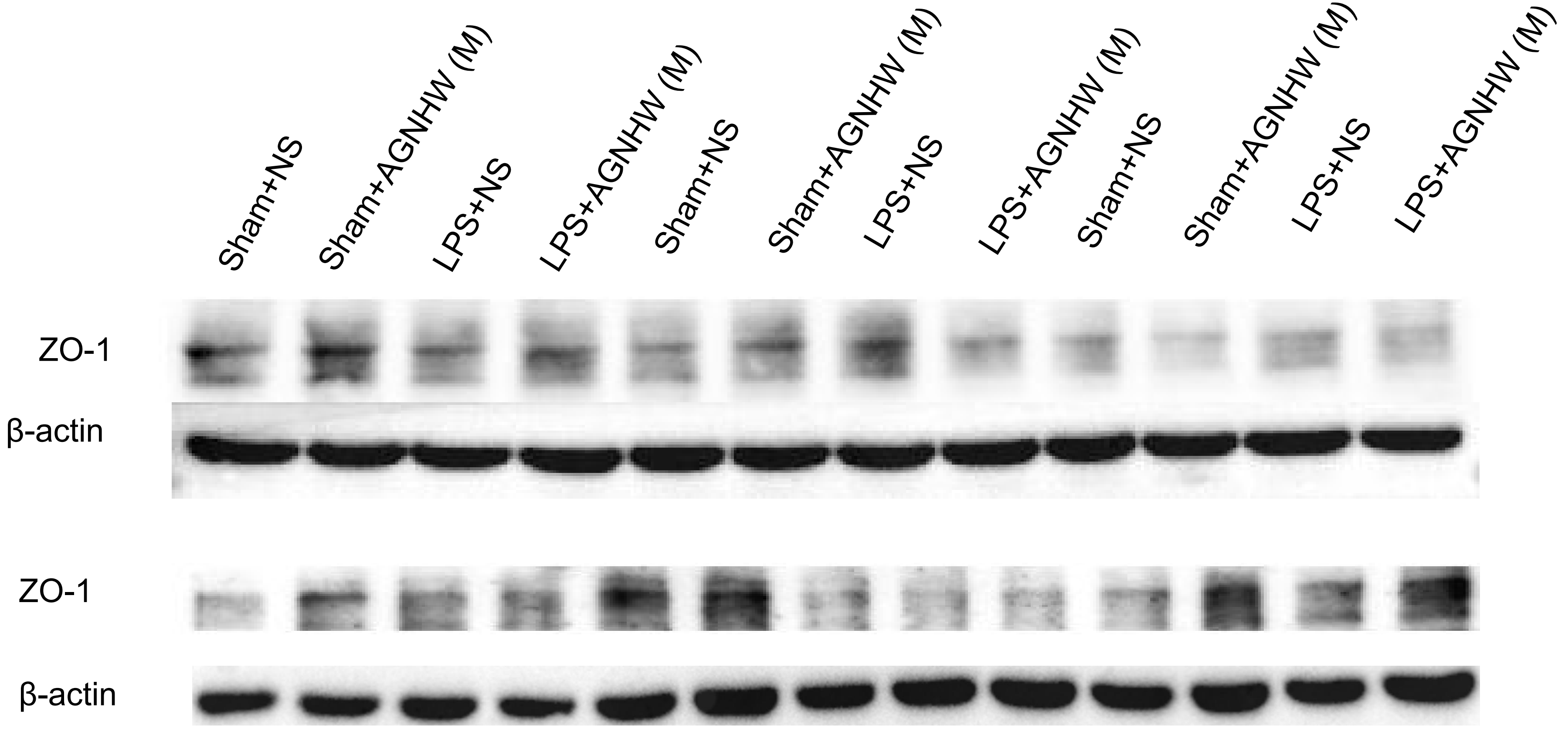
Sham+NS
Sham+AGNHW (M)
LPS+NS
LPS+AGNHW (M)
Sham+NS
Sham+AGNHW (M)
LPS+NS
LPS+AGNHW (M)
Sham+NS
Sham+AGNHW (M)
LPS+NS
LPS+AGNHW (M)
ZO-1
β-actin
ZO-1
β-actin

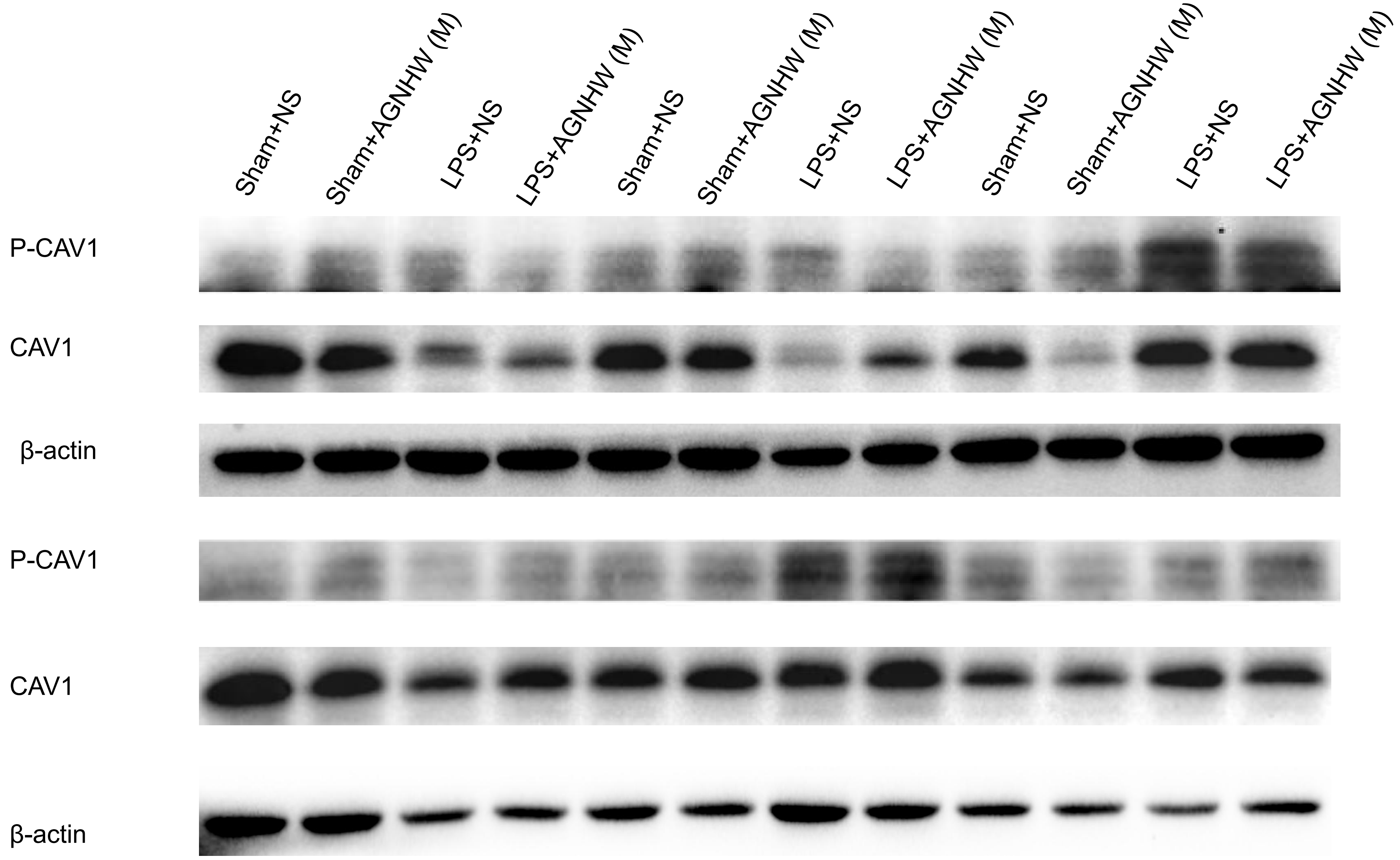
Sham+NS
Sham+AGNHW (M)
LPS+NS
LPS+AGNHW (M)
Sham+NS
Sham+AGNHW (M)
LPS+NS
LPS+AGNHW (M)
Sham+NS
Sham+AGNHW (M)
LPS+NS
LPS+AGNHW (M)
P-CAV1
CAV1
β-actin
P-CAV1
CAV1
β-actin

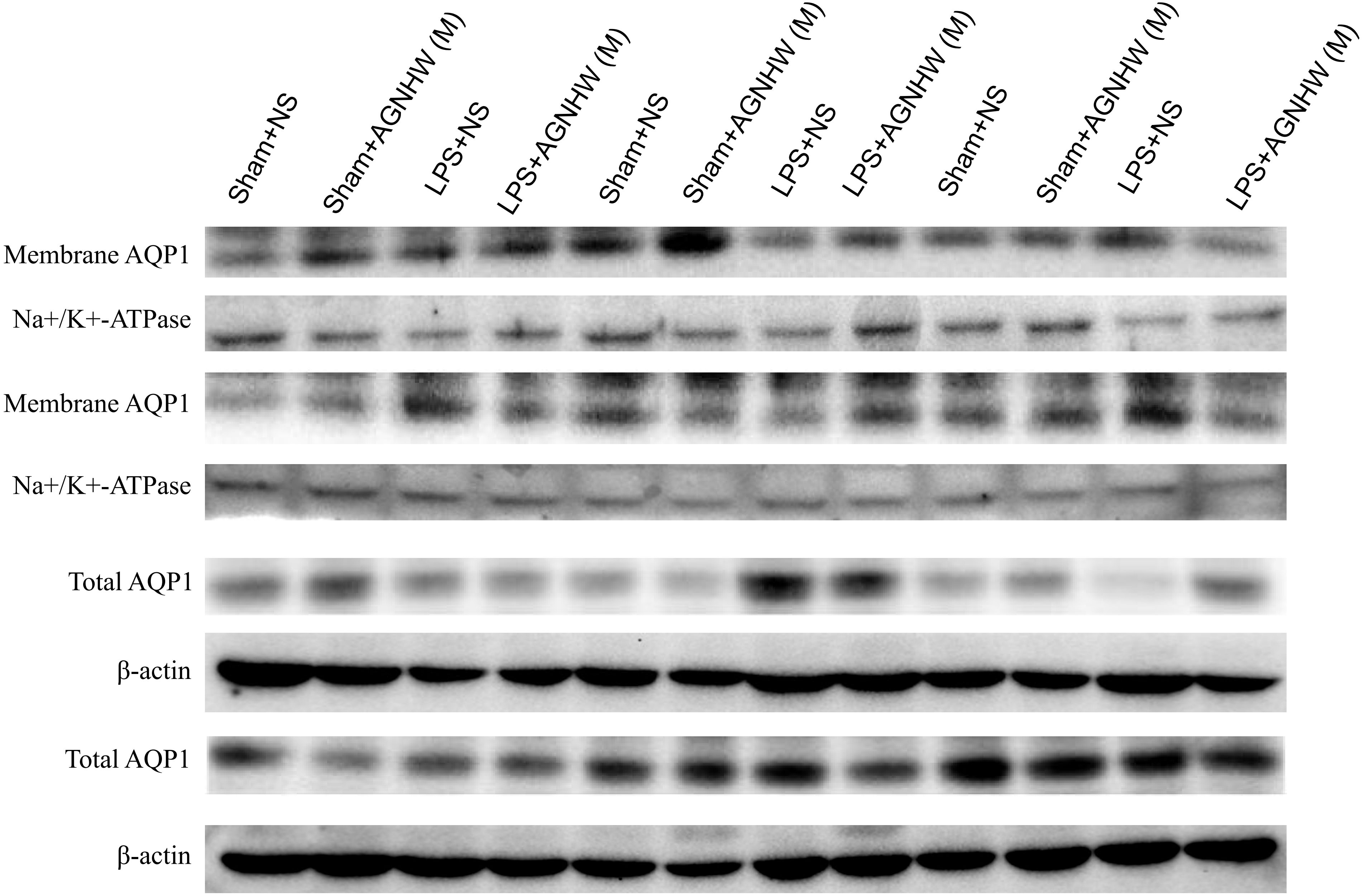
Sham+NS
Sham+AGNHW (M)
LPS+NS
LPS+AGNHW (M)
Sham+NS
Sham+AGNHW (M)
LPS+NS
LPS+AGNHW (M)
Sham+NS
Sham+AGNHW (M)
LPS+NS
LPS+AGNHW (M)
Membrane AQP1
Na+/K+-ATPase
Membrane AQP1
Na+/K+-ATPase
Total AQP1
β-actin
Total AQP1
β-actin

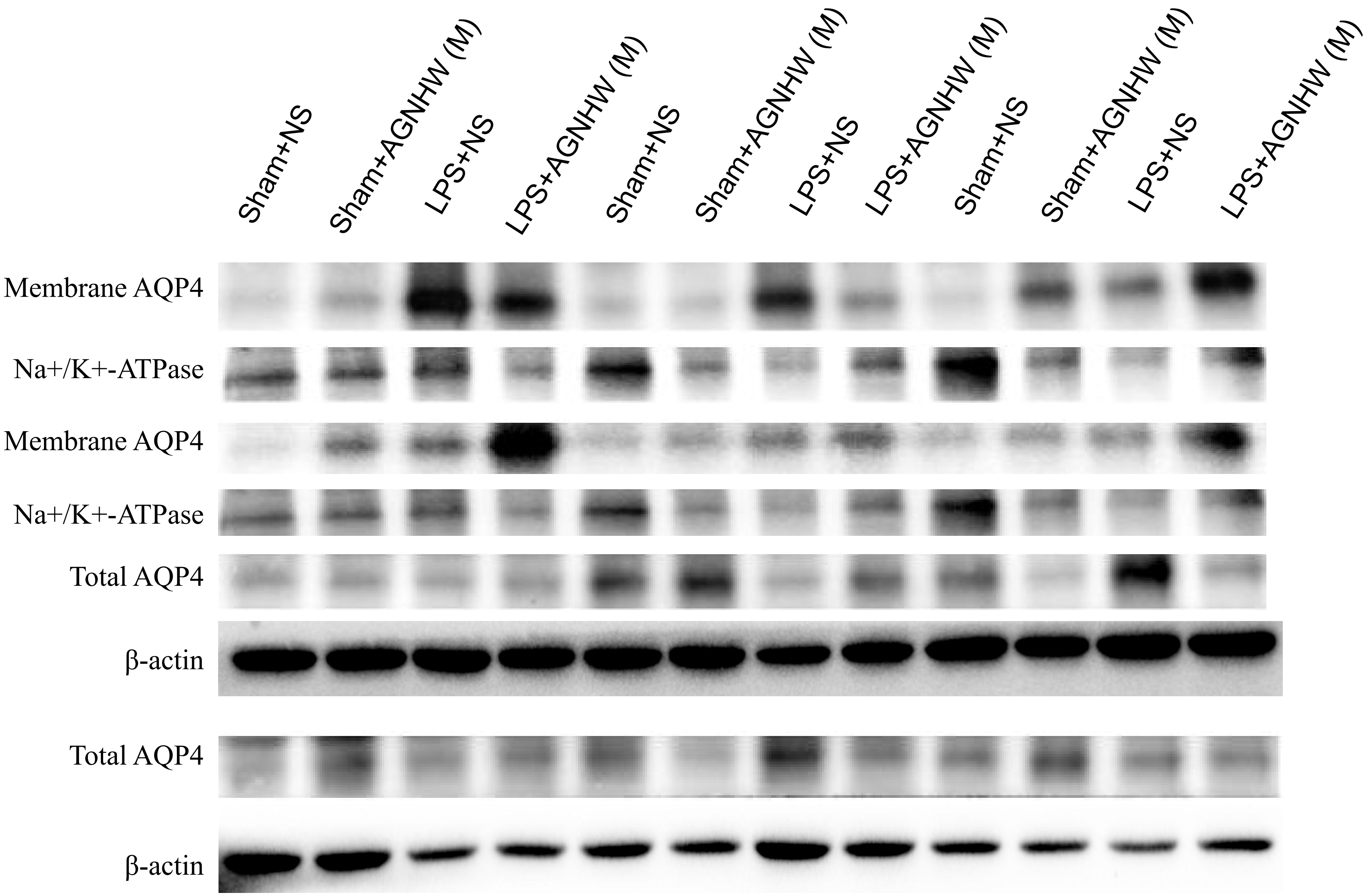
Sham+NS
Sham+AGNHW (M)
LPS+NS
LPS+AGNHW (M)
Sham+NS
Sham+AGNHW (M)
LPS+NS
LPS+AGNHW (M)
Sham+NS
Sham+AGNHW (M)
LPS+NS
LPS+AGNHW (M)
Membrane AQP4
Na+/K+-ATPase
Membrane AQP4
Na+/K+-ATPase
Total AQP4
β-actin
Total AQP4
β-actin

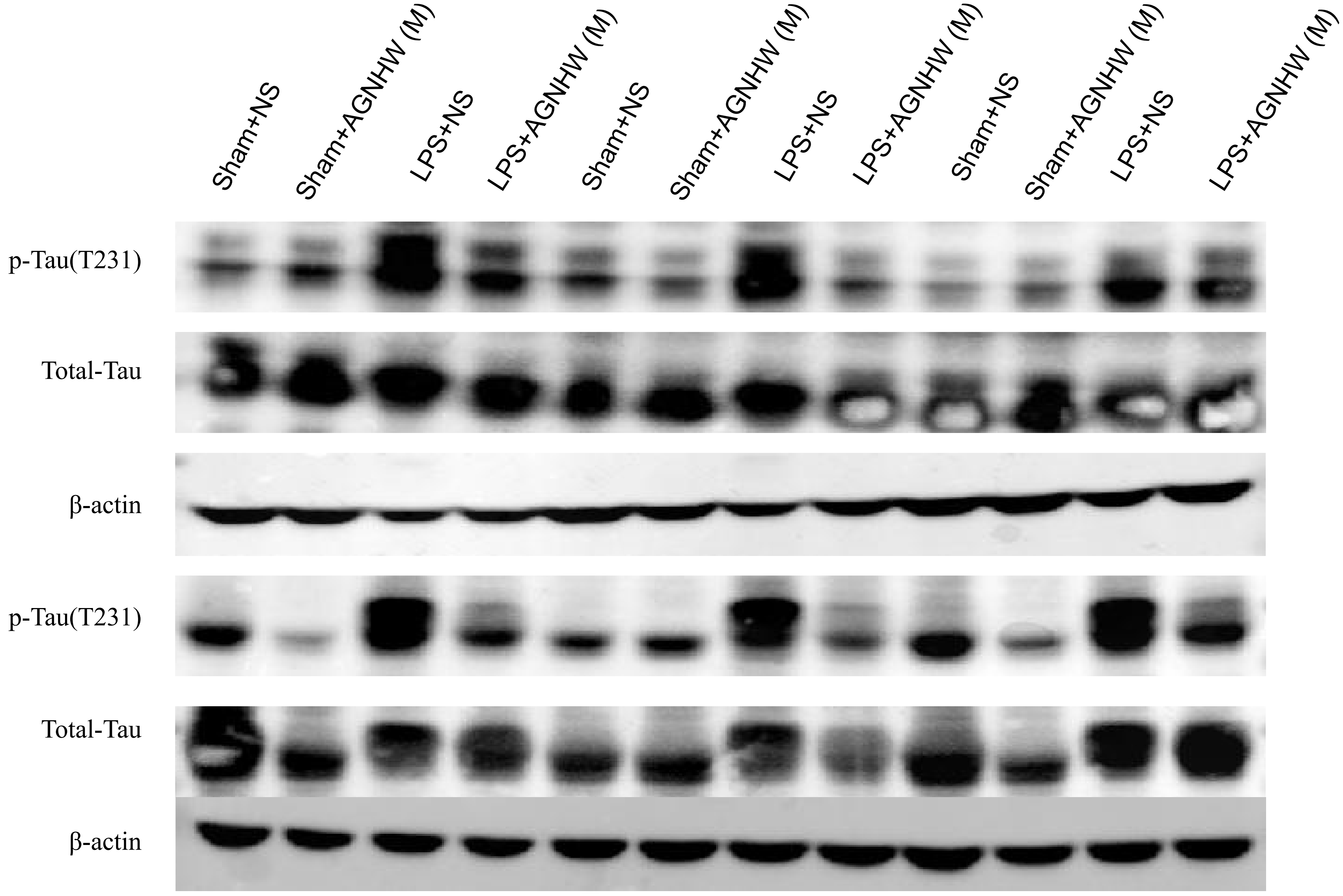
Sham+NS
Sham+AGNHW (M)
LPS+NS
LPS+AGNHW (M)
Sham+NS
Sham+AGNHW (M)
LPS+NS
LPS+AGNHW (M)
Sham+NS
Sham+AGNHW (M)
LPS+NS
LPS+AGNHW (M)
p-Tau(T231)
Total-Tau
β-actin
p-Tau(T231)
Total-Tau
β-actin

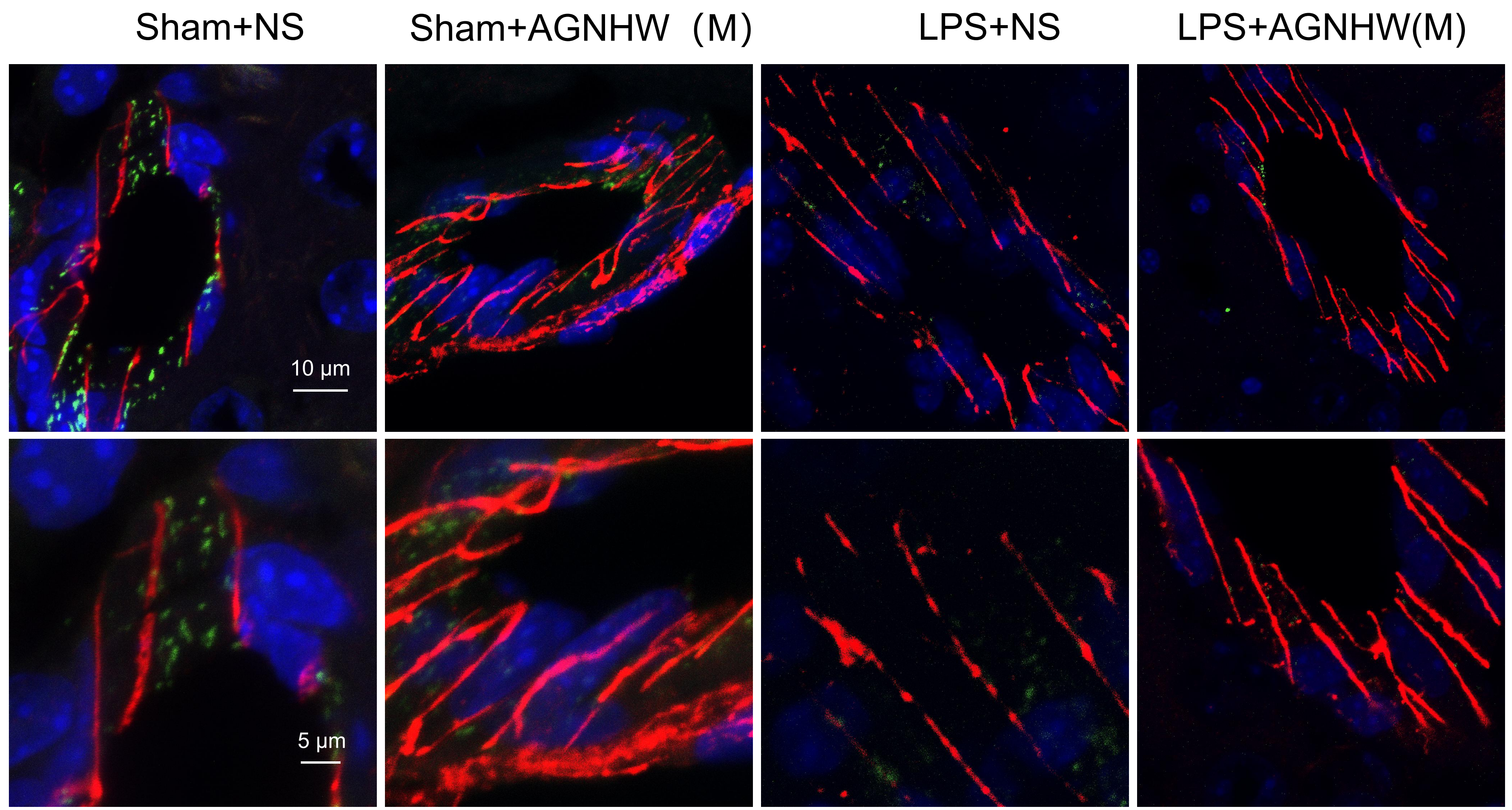
Sham+NS
Sham+AGNHW (M)
LPS+NS
LPS+AGNHW(M)
10 μm
5 μm

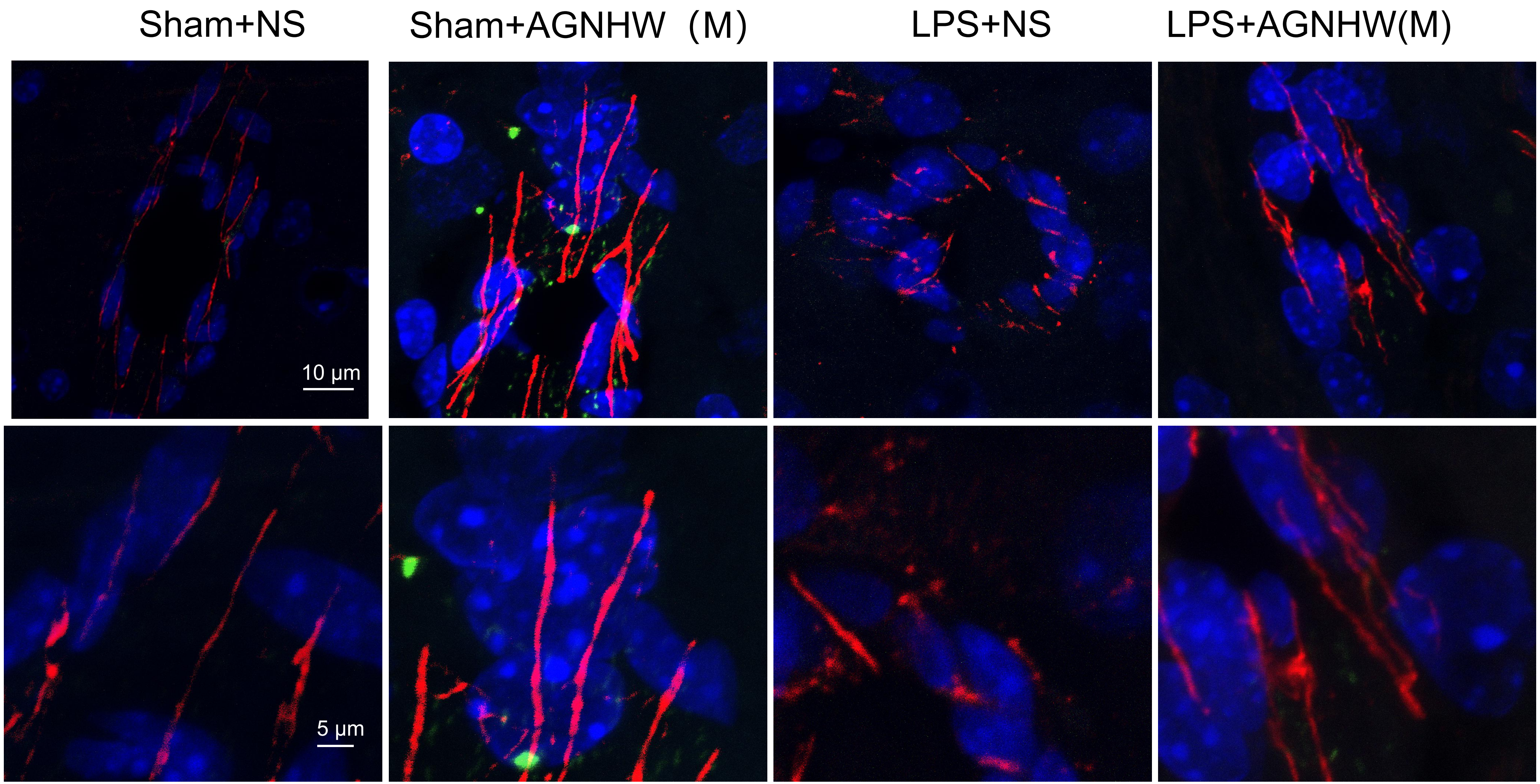

Sham+NS
Sham+AGNHW (M)
LPS+NS
LPS+AGNHW(M)
10 μm
5 μm

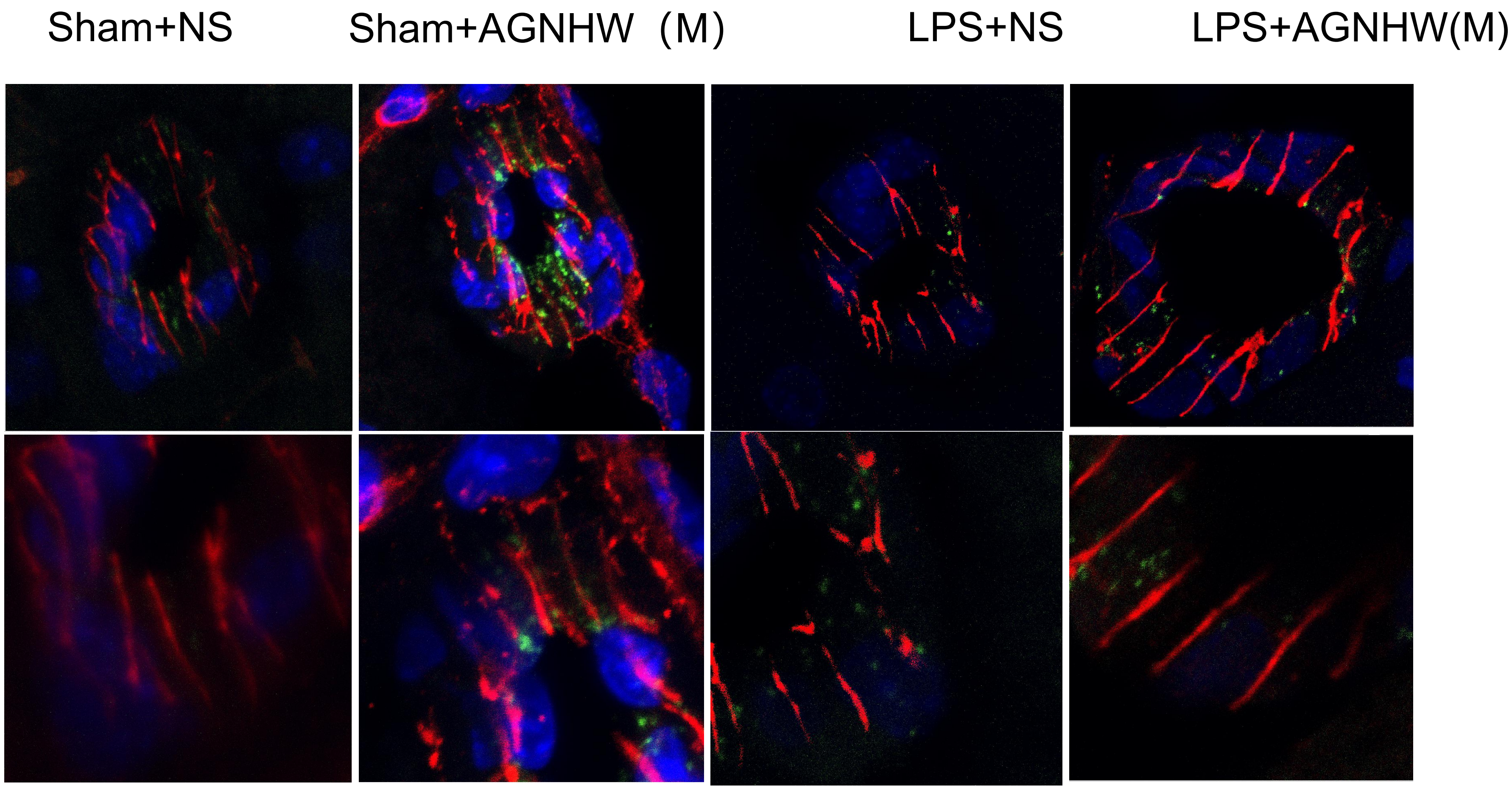
Sham+NS
Sham+AGNHW（M）
LPS+NS
LPS+AGNHW(M)

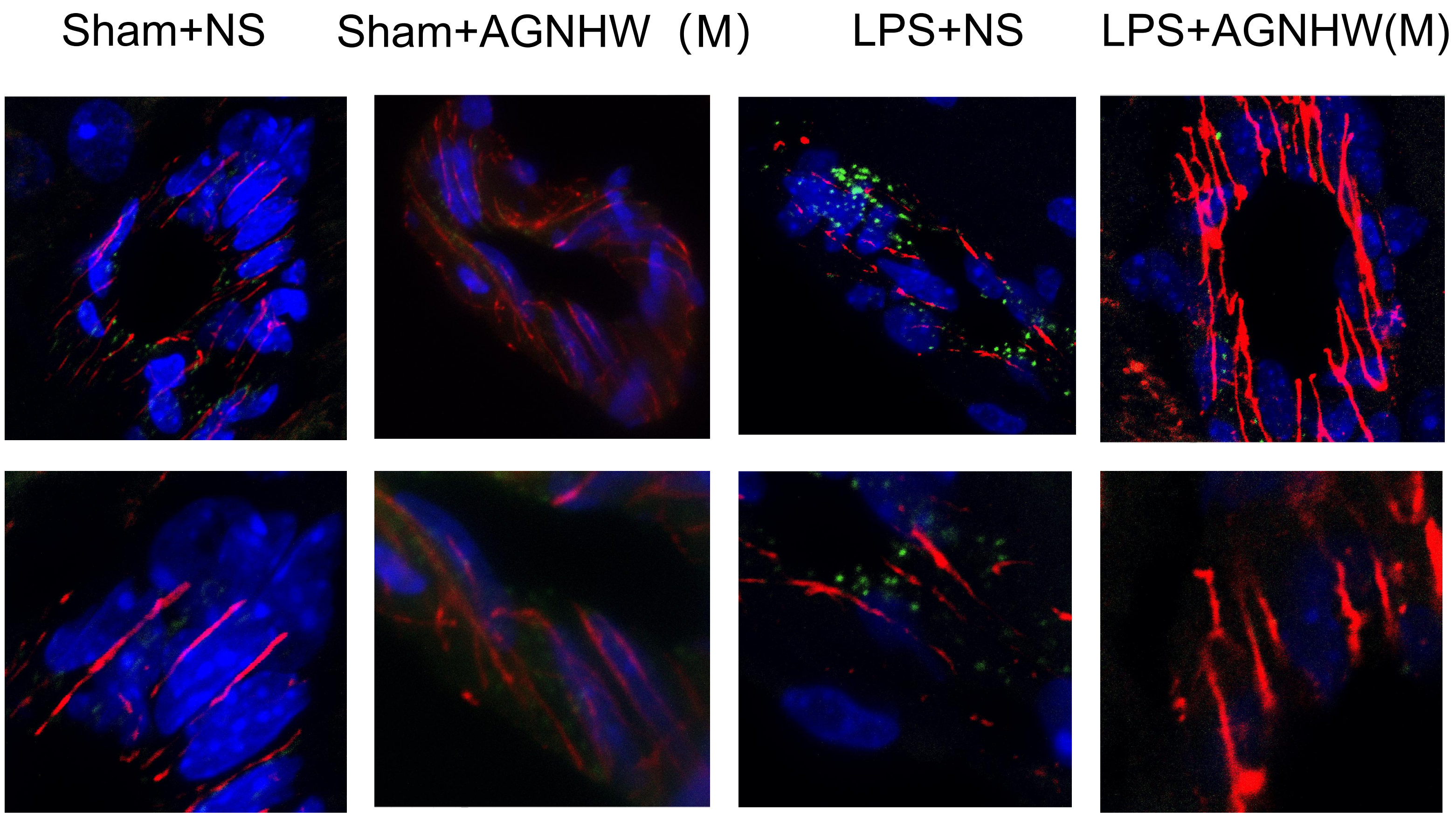
Sham+NS
Sham+AGNHW (M)
LPS+NS
LPS+AGNHW(M)

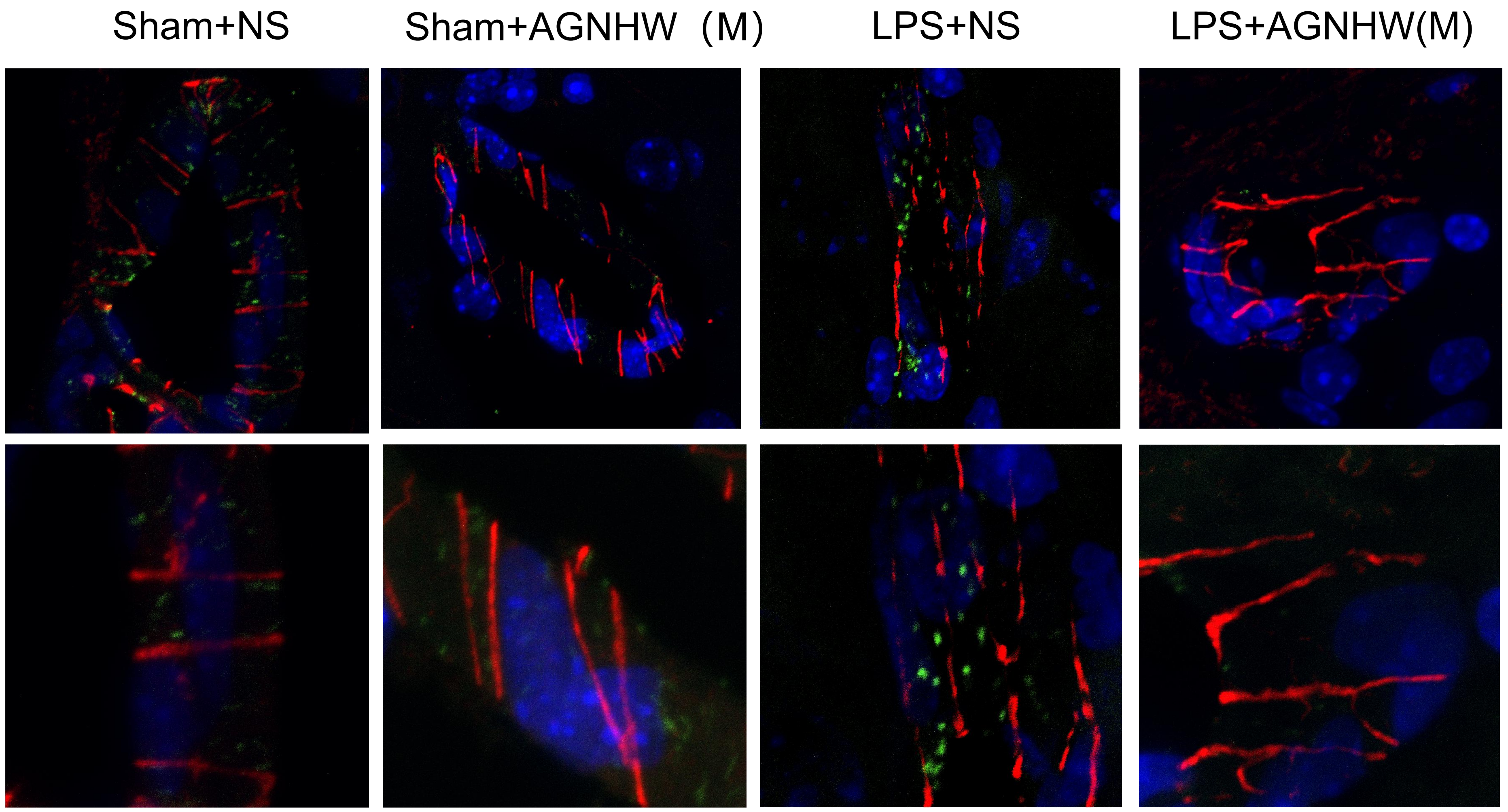
Sham+NS
Sham+AGNHW (M)
LPS+NS
LPS+AGNHW(M)

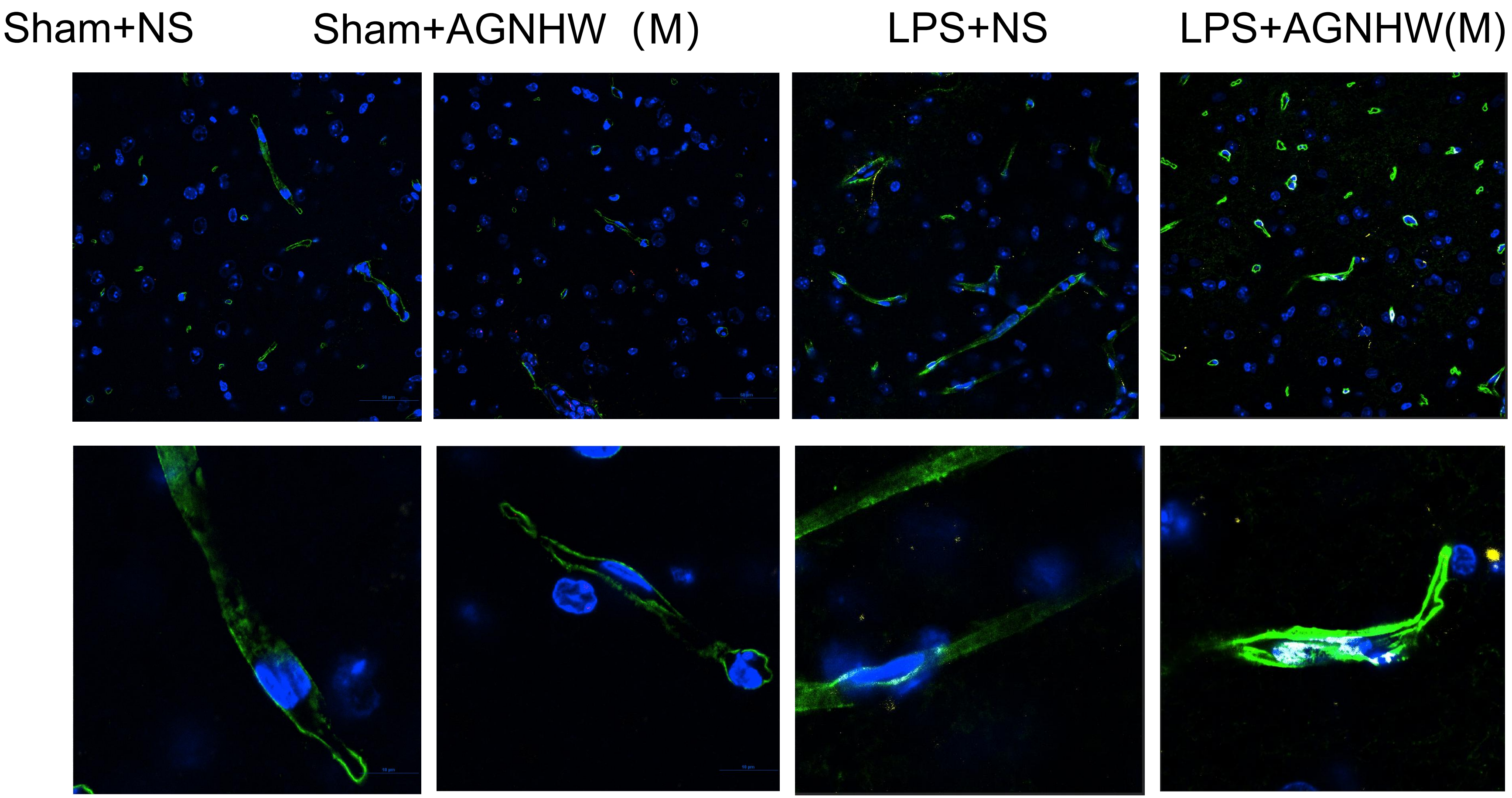
Sham+NS
Sham+AGNHW（M）
LPS+NS
LPS+AGNHW(M)

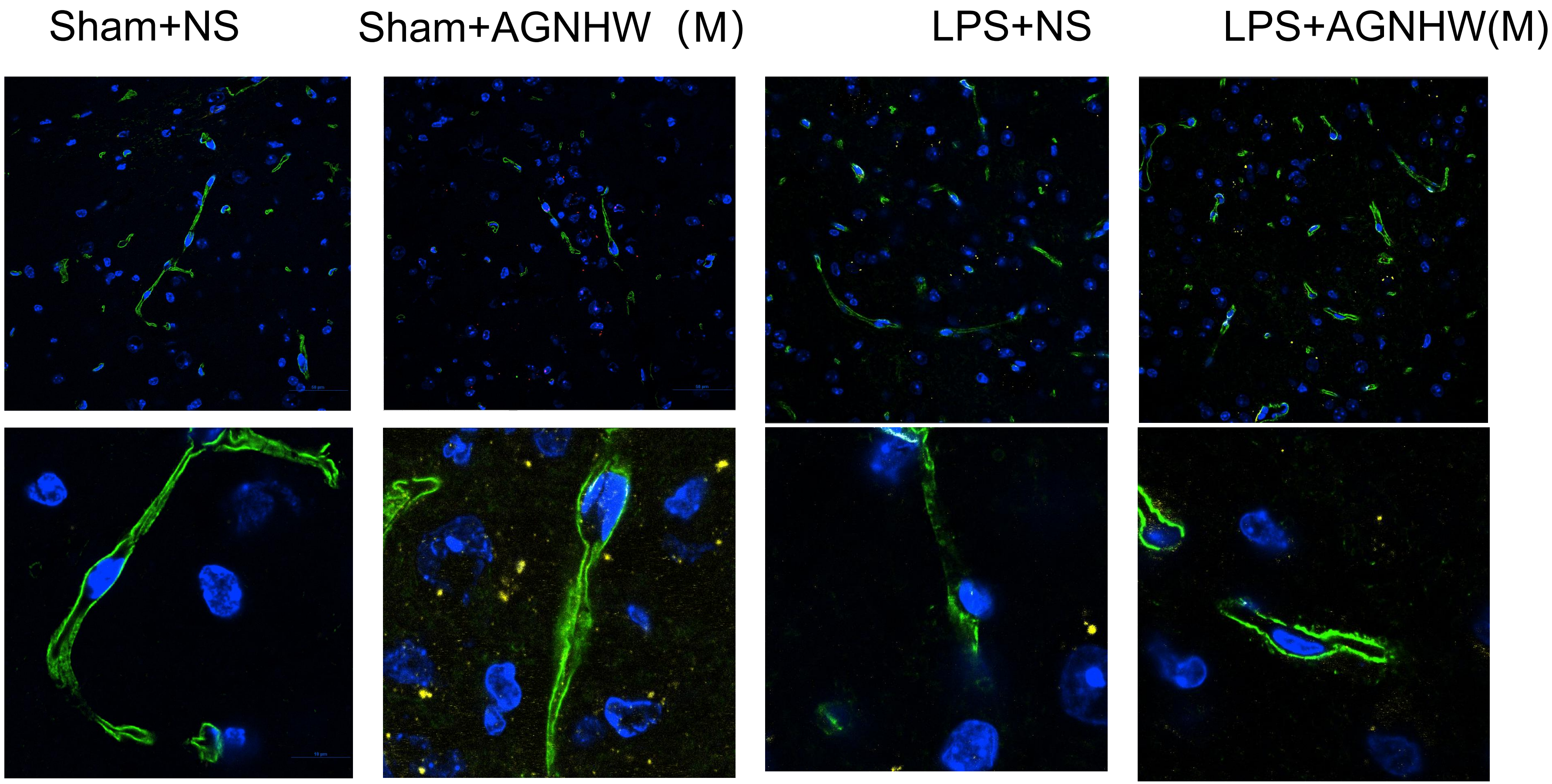
Sham+NS
Sham+AGNHW（M)
LPS+NS
LPS+AGNHW(M)

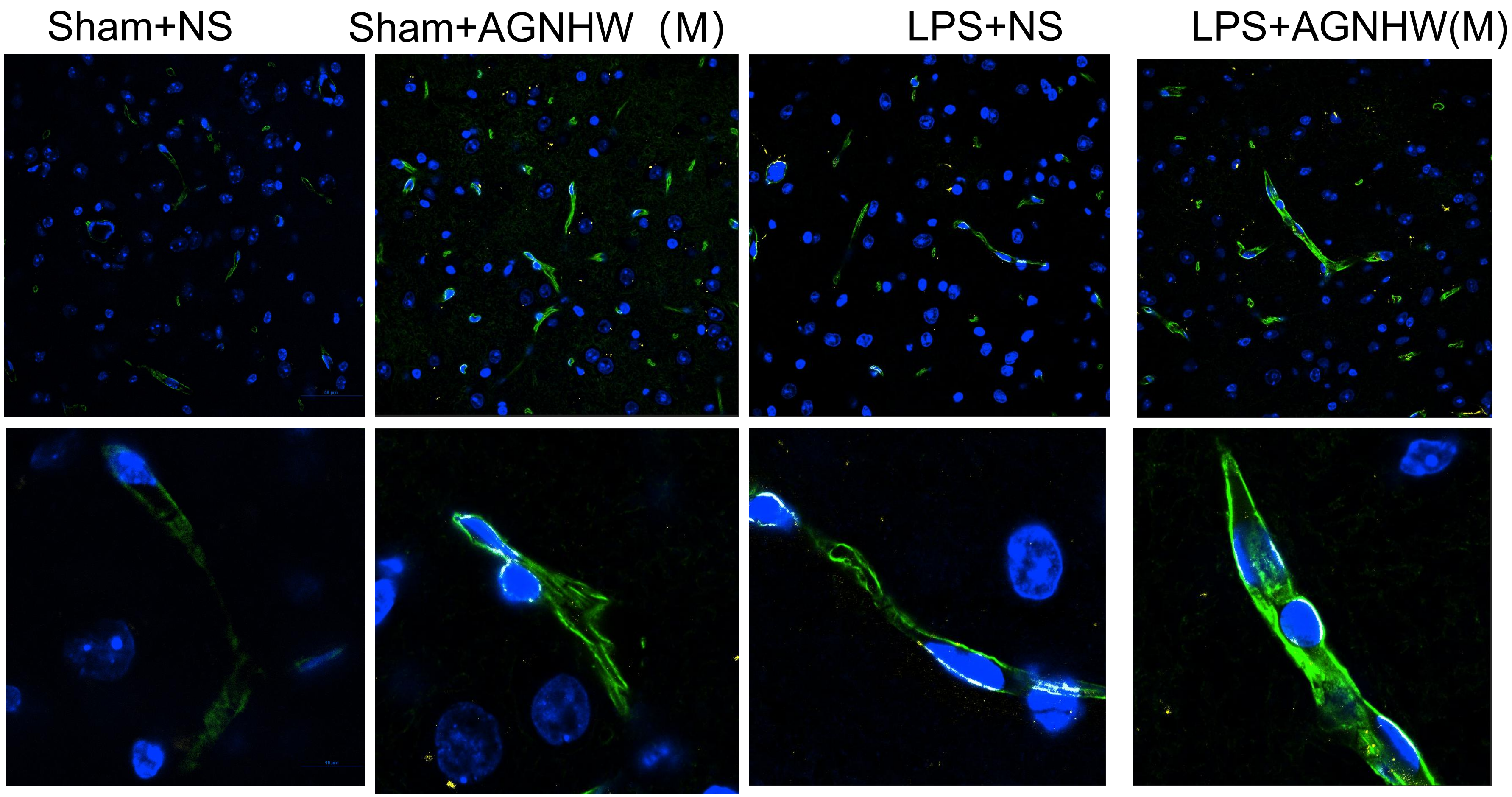
Sham+NS
Sham+AGNHW（M)
LPS+NS
LPS+AGNHW(M)

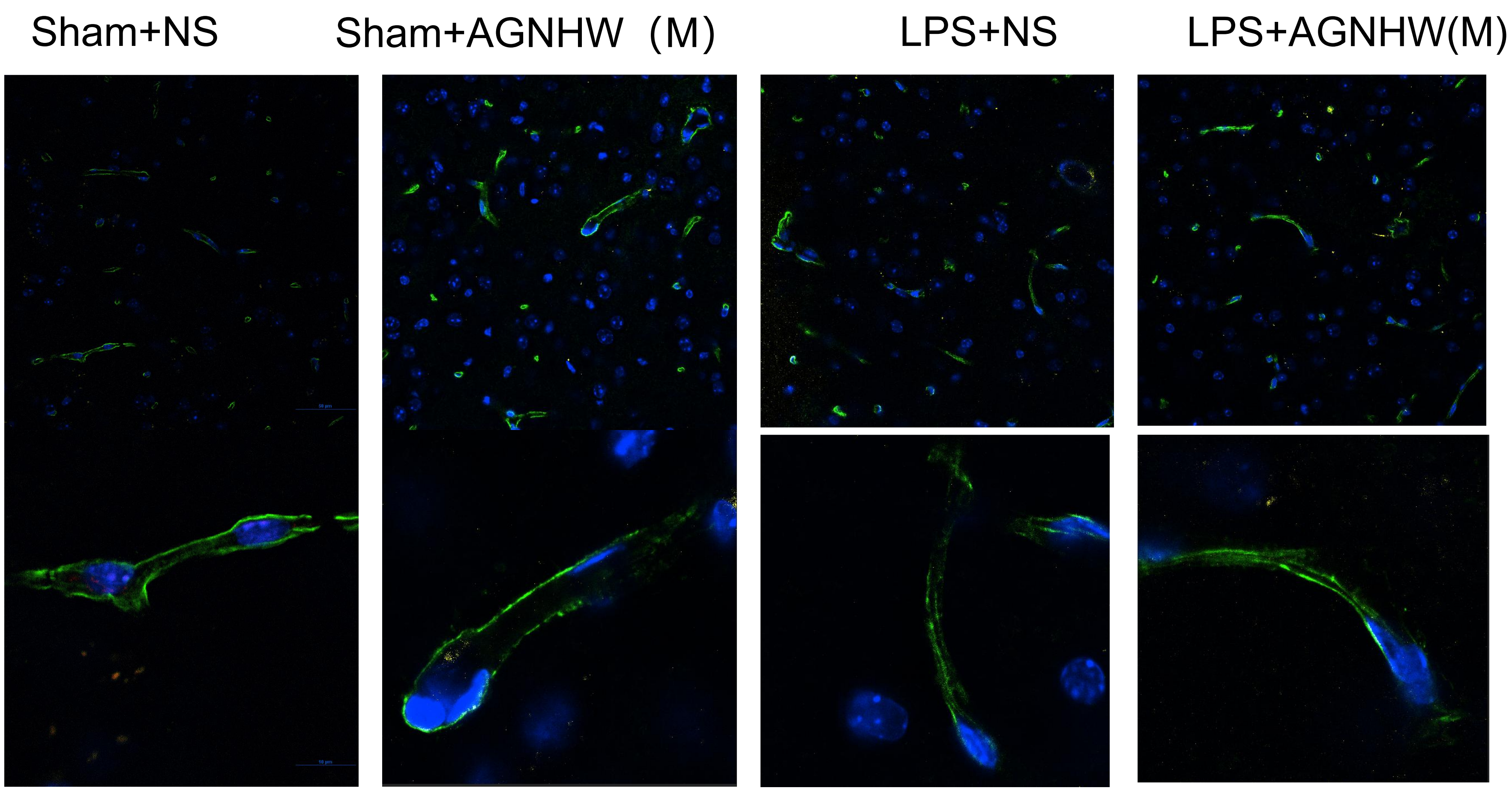
Sham+NS
Sham+AGNHW (M)
LPS+NS
LPS+AGNHW(M)

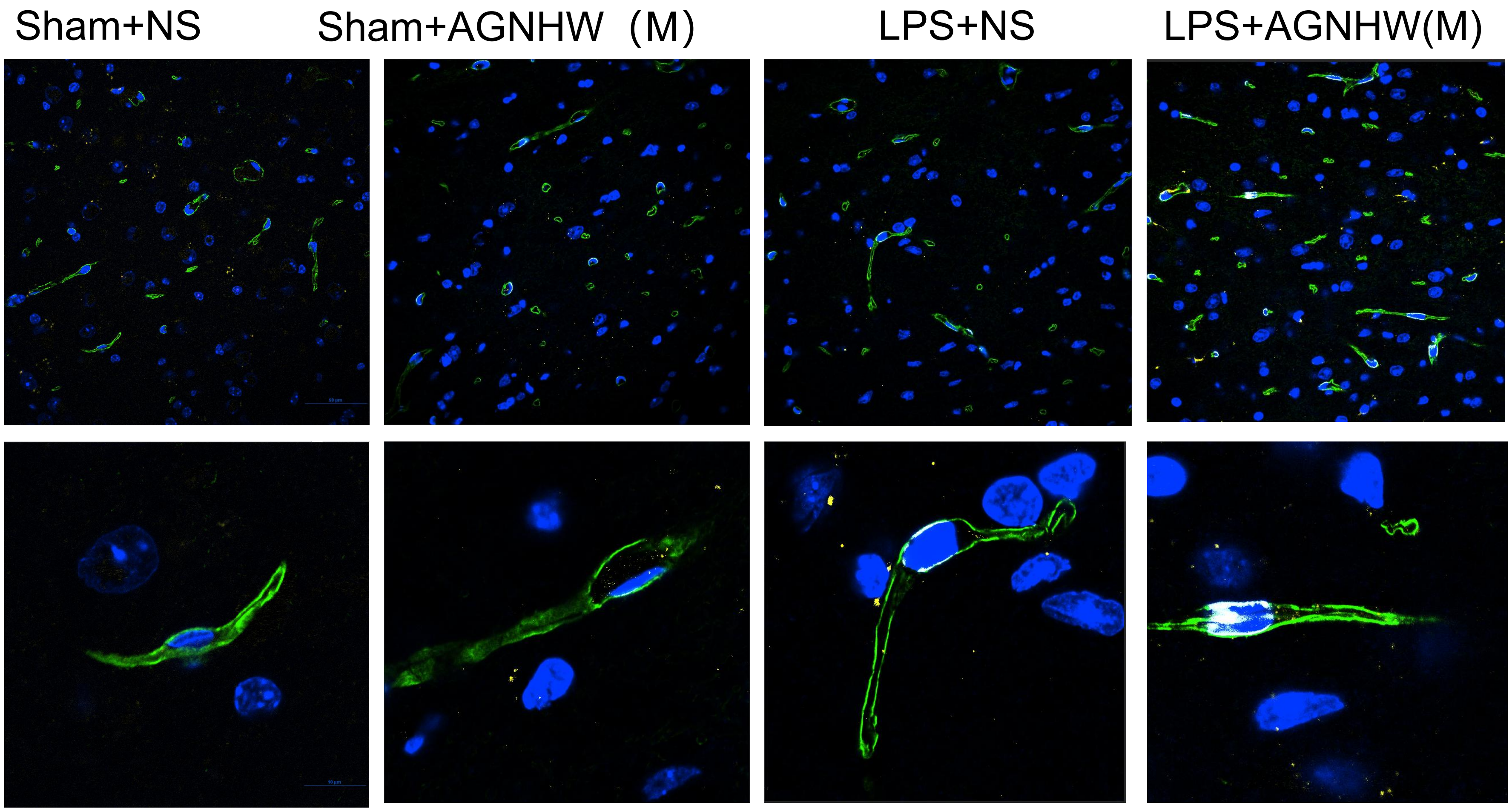
Sham+NS
Sham+AGNHW（M)
LPS+NS
LPS+AGNHW(M)

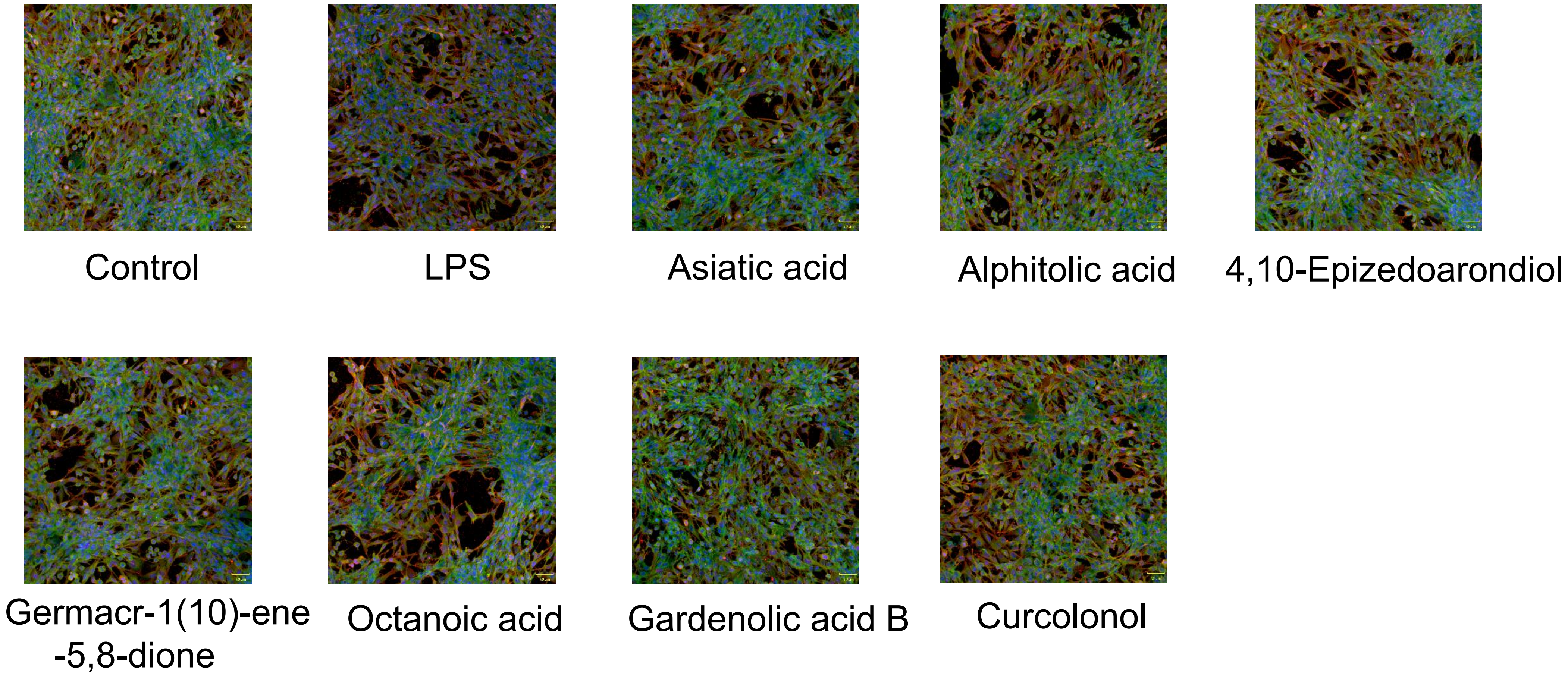
Control
LPS
Asiatic acid
Alphitolic acid
4,10-Epizedoarondiol
Germacr-1(10)-ene
-5,8-dione
Octanoic acid
Gardenolic acid B
Curcolonol

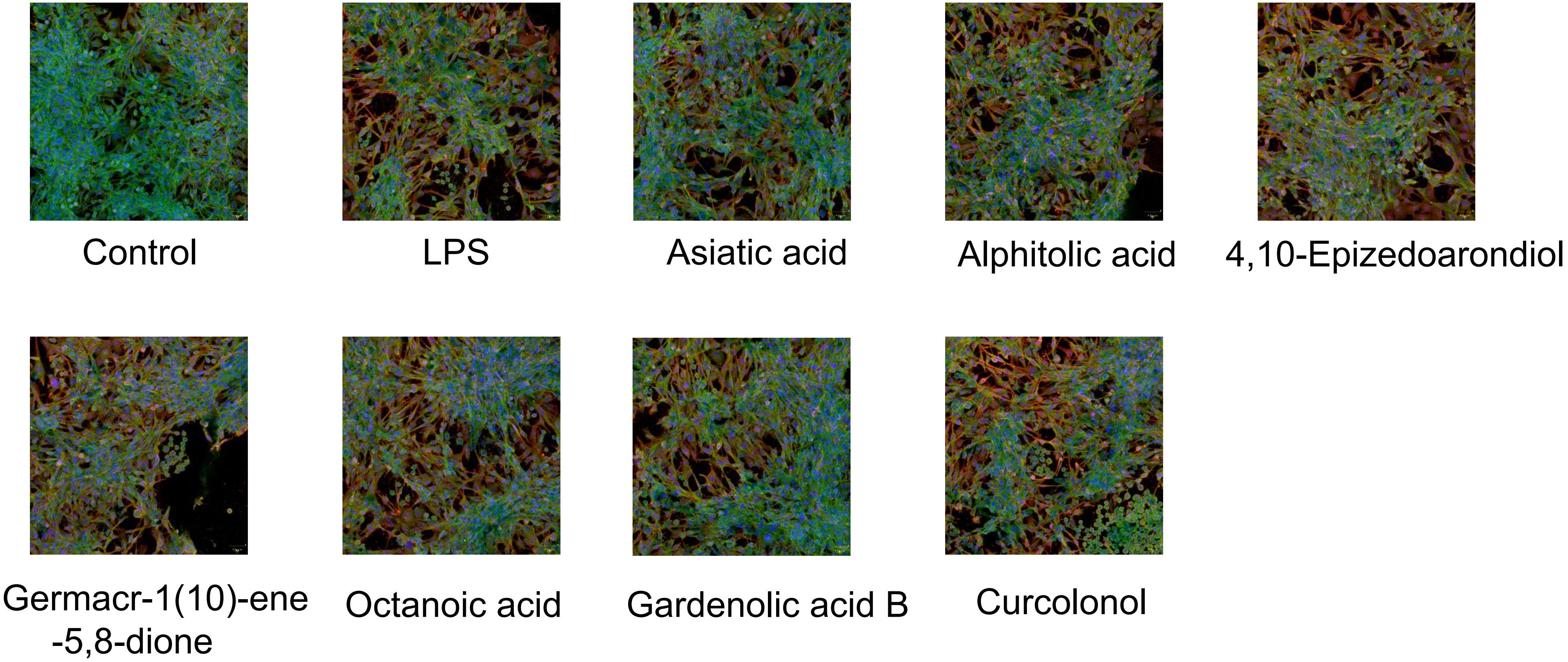
Control
LPS
Asiatic acid
Alphitolic acid
4,10-Epizedoarondiol
Germacr-1(10)-ene
-5,8-dione
Octanoic acid
Gardenolic acid B
Curcolonol

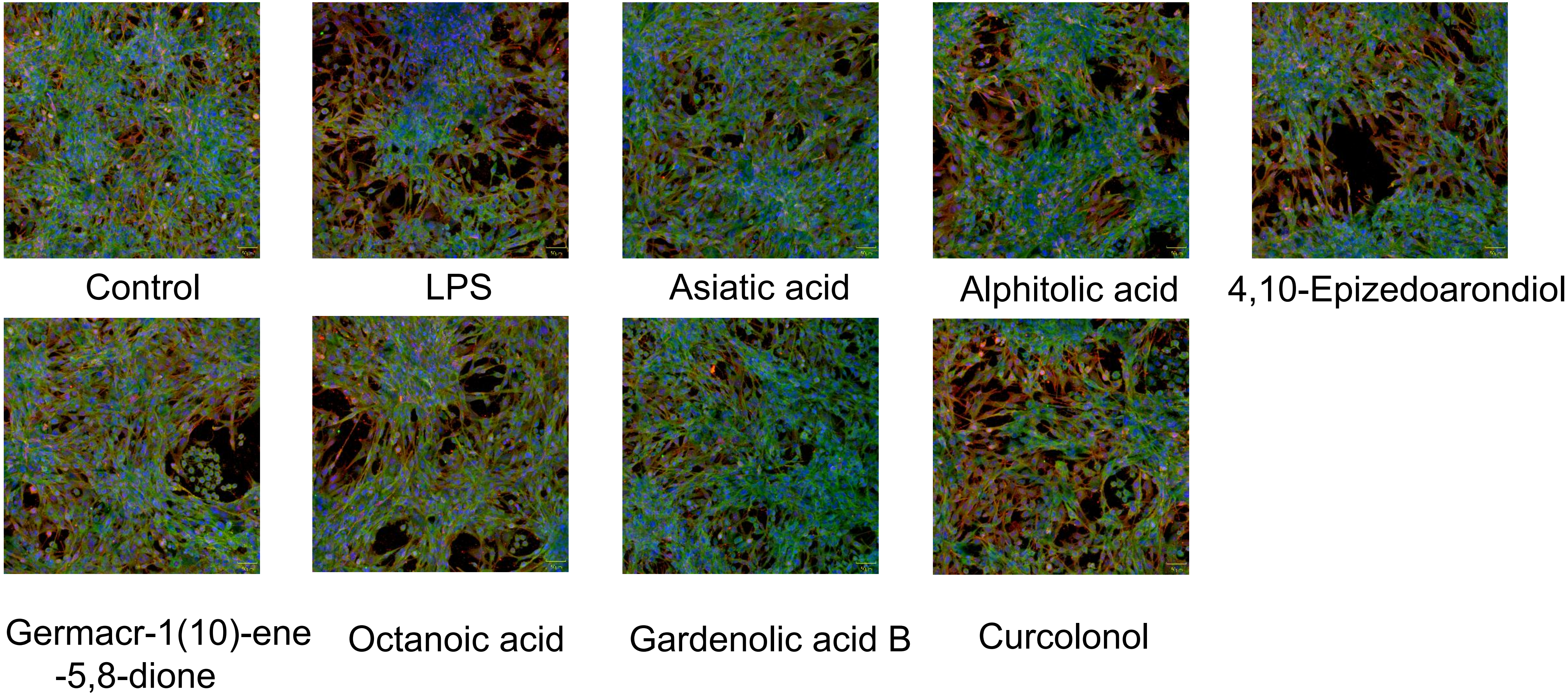
Control
LPS
Asiatic acid
Alphitolic acid
4,10-Epizedoarondiol
Germacr-1(10)-ene
-5,8-dione
Octanoic acid
Gardenolic acid B
Curcolonol

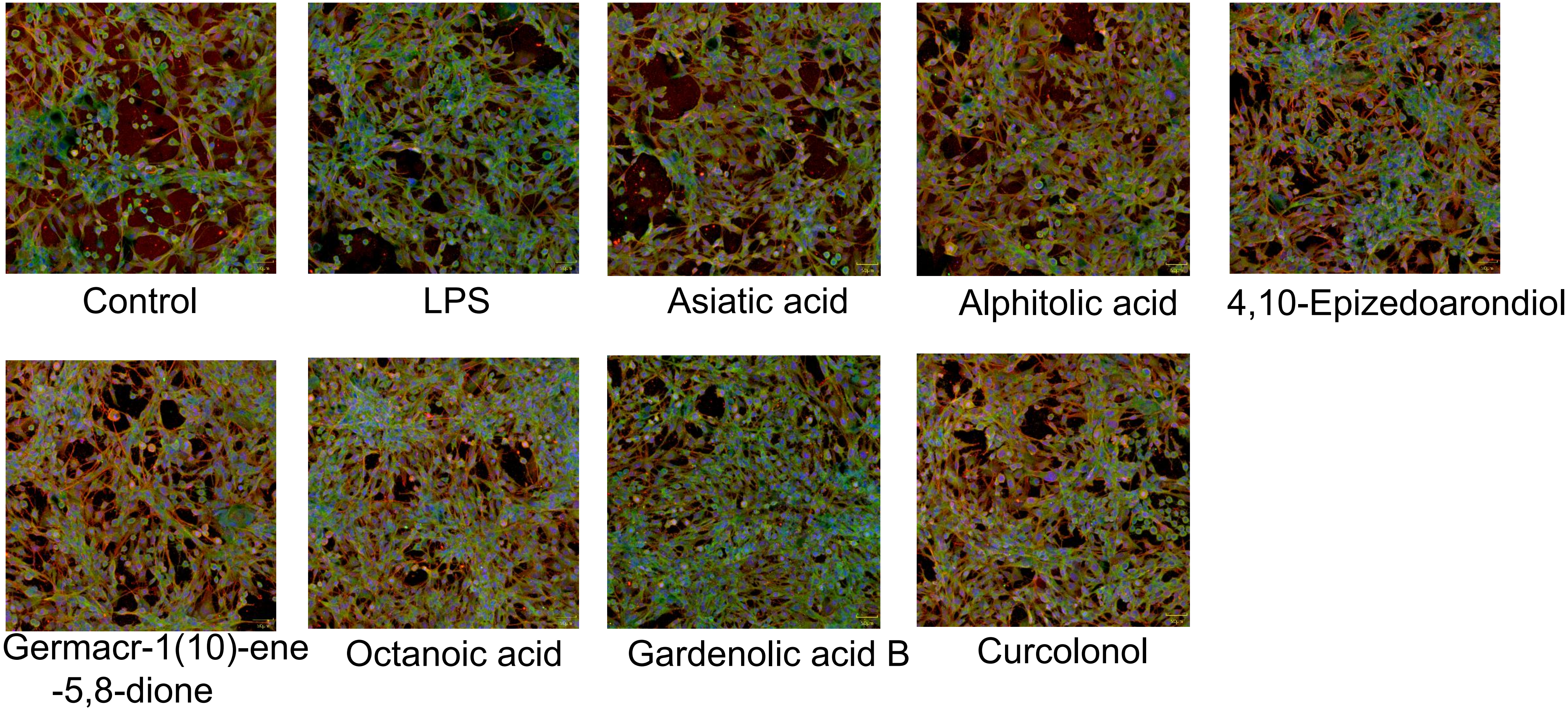
Control
LPS
Asiatic acid
Alphitolic acid
4,10-Epizedoarondiol
Germacr-1(10)-ene
-5,8-dione
Octanoic acid
Gardenolic acid B
Curcolonol

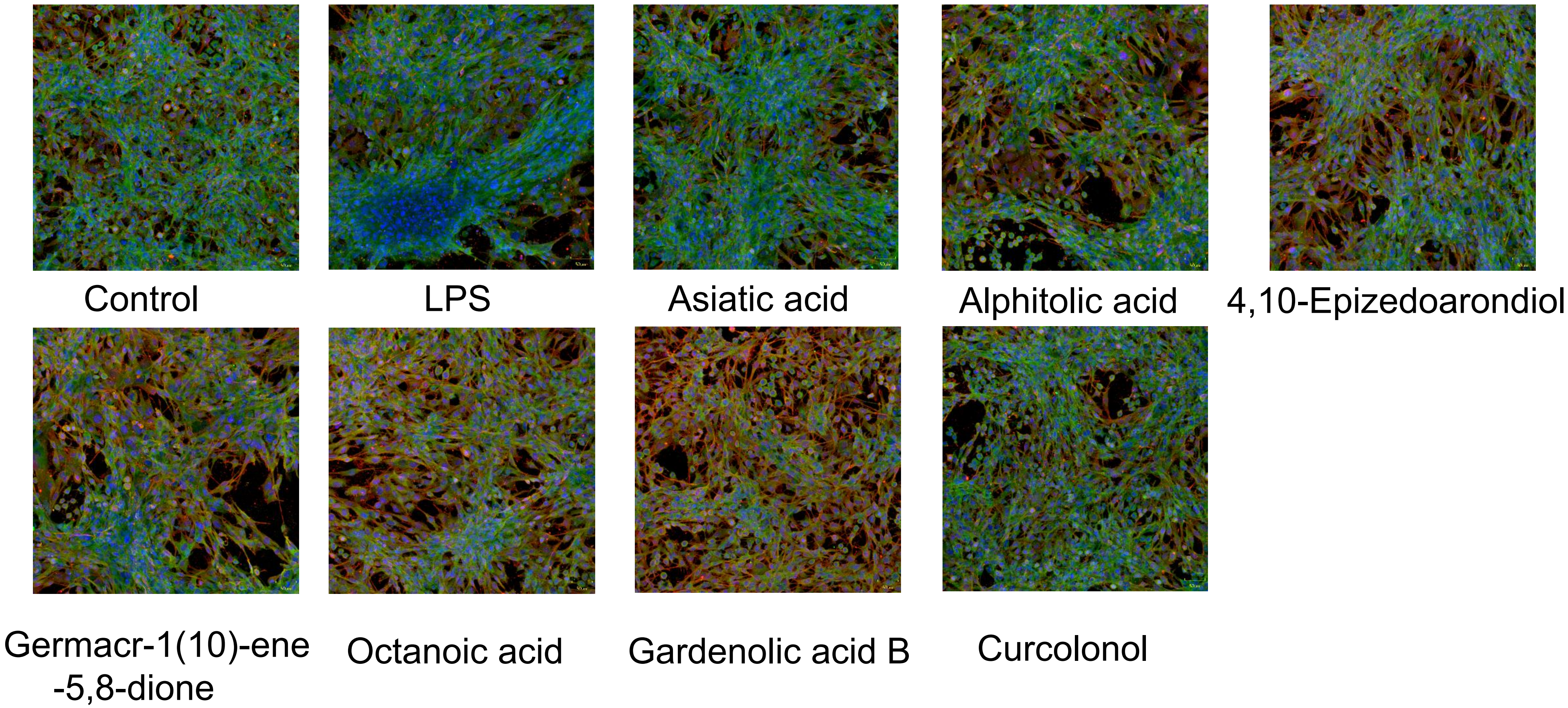
Control
LPS
Asiatic acid
Alphitolic acid
4,10-Epizedoarondiol
Germacr-1(10)-ene
-5,8-dione
Octanoic acid
Gardenolic acid B
Curcolonol

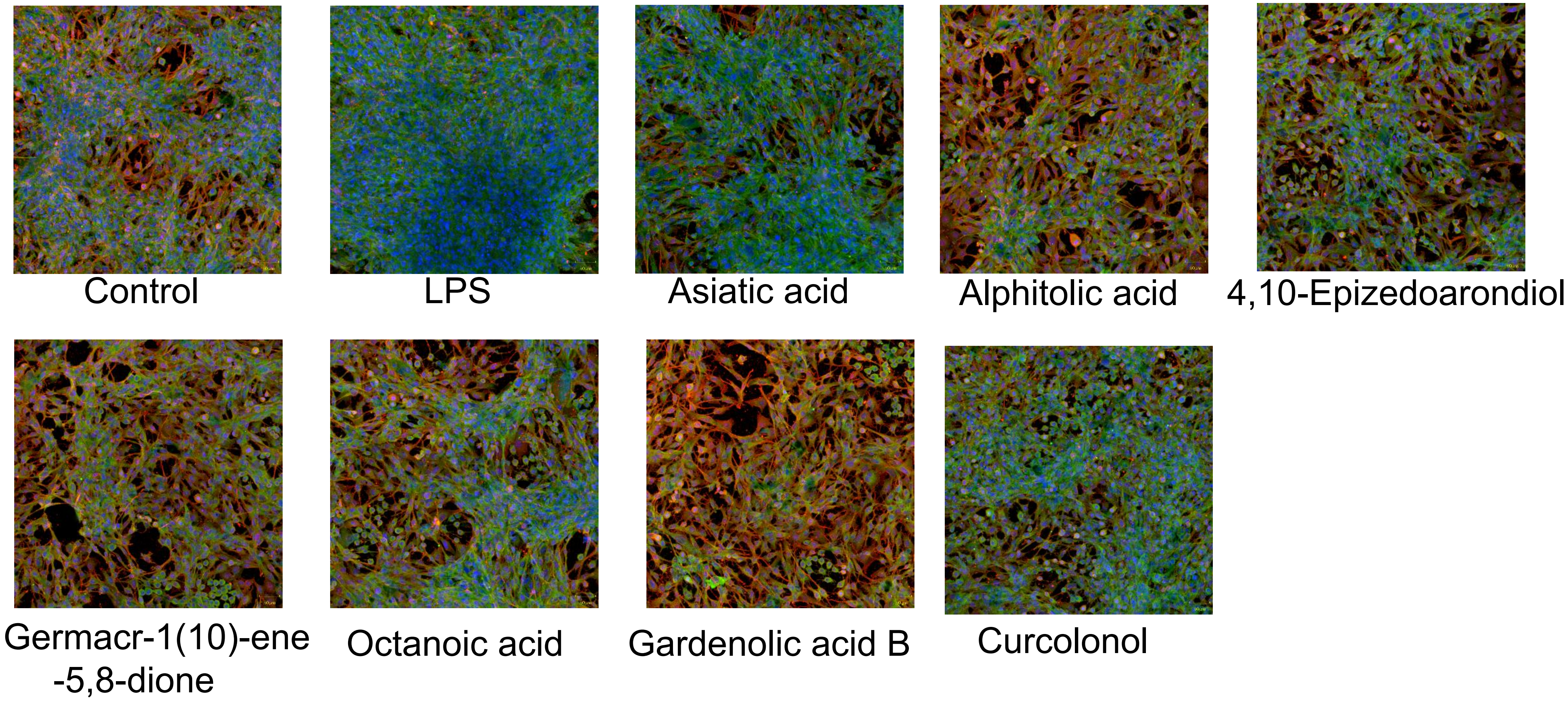
Control
LPS
Asiatic acid
Alphitolic acid
4,10-Epizedoarondiol
Germacr-1(10)-ene
-5,8-dione
Octanoic acid
Gardenolic acid B
Curcolonol
